# Starch synthase 4 is essential for coordination of starch granule formation with chloroplast division during Arabidopsis leaf expansion

**DOI:** 10.1111/nph.12455

**Published:** 2013-08-19

**Authors:** Matilda Crumpton-Taylor, Marilyn Pike, Kuan-Jen Lu, Christopher M Hylton, Regina Feil, Simona Eicke, John E Lunn, Samuel C Zeeman, Alison M Smith

**Affiliations:** 1 John Innes Centre, Norwich Research ParkNorwich, NR4 7UH, UK; 2 Institute of Agricultural Sciences, Eidgenössische Hochschule ZürichUniversitätstr. 2, 8092, Zürich, Switzerland; 3 Max-Planck Institute of Molecular Plant Physiology, Wissenschaftspark GolmAm Mühlenberg 1, 14476, Potsdam-Golm, Germany

**Keywords:** ADPglucose, *Arabidopsis thaliana*, chloroplast, leaf expansion, starch granule, starch synthase, starch synthesis

## Abstract

- *Arabidopsis thaliana* mutants lacking the SS4 isoform of starch synthase have strongly reduced numbers of starch granules per chloroplast, suggesting that SS4 is necessary for the normal generation of starch granules. To establish whether it plays a direct role in this process, we investigated the circumstances in which granules are formed in *ss4* mutants.
- Starch granule numbers and distribution and the accumulation of starch synthase substrates and products were investigated during *ss4* leaf development, and in *ss4* mutants carrying mutations or transgenes that affect starch turnover or chloroplast volume.
- We found that immature *ss4* leaves have no starch granules, but accumulate high concentrations of the starch synthase substrate ADPglucose. Granule numbers are partially restored by elevating the capacity for glucan synthesis (via expression of bacterial glycogen synthase) or by increasing the volumes of individual chloroplasts (via introduction of *arc* mutations). However, these granules are abnormal in distribution, size and shape.
- SS4 is an essential component of a mechanism that coordinates granule formation with chloroplast division during leaf expansion and determines the abundance and the flattened, discoid shape of leaf starch granules.

## Introduction

The process by which starch granules arise is not known. Suggestions range from largely physico-chemical mechanisms (Doi, 1965; Geddes & Greenwood, 1969; Ziegler *et al.*, 2005) to the existence of specific protein primers analogous to the glycogenins of fungi and animals (e.g. Rothschild & Tandecarz, 1994; Singh *et al.*, 1995; Langeveld *et al.*, 2002; Chatterjee *et al.*, 2005). Recent attention has focussed on the role of one isoform of soluble starch synthase, starch synthase 4 (SS4, At4 g18240). Although SS4 contributes little to total starch synthase activity, *ss4* mutants of Arabidopsis have at most one or two starch granules per chloroplast (Roldán *et al.*, 2007) rather than the normal five or six (Crumpton-Taylor *et al.*, 2012). No other starch synthase is individually necessary for normal granule numbers (Roldán *et al.*, 2007), thus SS4 may have a specific function in granule formation. However, other isoforms of starch synthase may partially substitute for this function. The additional loss of SS3 further reduces starch granule numbers in the *ss4* mutant background (Szydlowski *et al.*, 2009; Mérida & D′Hulst, 2012).

The importance of SS4 for starch granule formation remains to be established. First, it is not known whether SS4 is required primarily for maintenance of starch granule numbers in mature leaves, or whether it also has a role in immature leaves where new granules arise in concert with chloroplast division (Crumpton-Taylor *et al.*, 2012). Second, it is not clear whether the reduction in starch granule numbers in *ss4* mutants is a direct or an indirect consequence of the loss of SS4. Mutants have several additional phenotypes including reduced growth rates, altered starch granule anatomy and morphology and a reduction in the extent of diel starch turnover (Roldán *et al.*, 2007). It remains possible that the reduction in granule numbers in *ss4* mutants is an indirect consequence of one of these alterations. Third, a recent study suggests that SS4 may be limiting for starch synthesis in wild-type plants. Its overexpression reportedly results in higher concentrations of starch at the end of the day and accelerated plant growth (Gámez-Arjona *et al.*, 2011). These results have important implications for the control of starch turnover and are of biotechnological interest, but the relationship between starch concentrations and starch granule numbers and sizes in plants with elevated SS4 was not reported.

The aim of our work was to establish whether SS4 has a direct or an indirect role in starch granule formation, and to shed further light on where and when its actions are required for the establishment of normal granule numbers. To this end we examined the phenotype of the *ss4* mutant through leaf development, and investigated the impact of loss of SS4 in mutant and transgenic backgrounds in which starch metabolism is altered or chloroplast volumes are abnormally large. Our results indicate that SS4 is directly and specifically required for the establishment of normal numbers and distributions of starch granules during leaf expansion, and that it is also necessary for the normal flattened, discoid shape of leaf starch granules.

## Materials and Methods

### Plant material

*Arabidopsis thaliana* plants were grown in compost at 20°C with 12 h light (200 μmol photons m^−2^ s^−1^), 12 h dark and were used as mature, nonflowering rosettes unless otherwise stated. For examination of roots, plants were grown on Phytogel with nutrients (Haughn & Somerville, 1986). The *ss4-1* mutant and the double mutant *ss3ss4* were described in Roldán *et al.* (2007) and Szydlowski *et al*. (2009), respectively. The *ss4-3* mutant (SALK_096130) was used unless otherwise stated. It carries a T-DNA insertion in intron 4 of the *SS4* gene, + 2186 bp from the start codon. The *sex1-8* (*gwd*) mutant (SALK_077211) was described in Ritte *et al*. (2006). It carries a T-DNA insertion in an intron of *GWD*, lacks detectable GWD protein, and is phenotypically indistinguishable from the null mutant *sex1-3* (Yu *et al.*, 2001). *arc* mutants were: *arc3-2* (SALK_057144), *arc5-2* (SAIL_71_D11), *arc6-5* (SAIL_693_G04) and *arc10-2* (SALK_073878) (Crumpton-Taylor *et al.*, 2012). Double mutants were identified in F_2_ populations derived from crosses by PCR on genomic DNA, using the oligonucleotide primers listed in Supporting Information Table S1.

### Complementation of the *ss4* mutant

A full-length *SS4* cDNA was introduced into the destination vector pK7FWG2,0 (Karimi *et al.*, 2002) by Gateway® LR clonase™ II (Invitrogen) recombination, under the control of a 35S promoter and upstream of *eGPF* present in the vector. The resulting binary vector was introduced into *Agrobacterium tumefaciens* strain GV3101 and used to transform *ss4-3* plants. Transformants were selected on media containing kanamycin. Presence of the transgene was confirmed using the oligonucleotide primers shown in Table S1 and homozygous lines were developed.

### Generation of lines expressing *Agrobacterium glgA*

The full-length coding sequence of glycogen synthase *glgA* was amplified from genomic DNA of *A. tumefaciens* strain GV3101 and cloned into pDONR 221 via Gateway® BP clonase™ II (Invitrogen) recombination. Using Gateway® LR clonase™ II recombination, *glgA* was transferred into the binary vector pB7WGY2, modified to contain a chloroplast targeted transit peptide (cTP) sequence (the first 53 amino acids of the Rubisco small subunit; At5 g38430) upstream and in frame with the N-terminal YFP (Fig. S9a). The resulting *GS*-containing construct (YFP-GS) was transformed into *ss3ss4* double mutant plants. Four independent GS-transformed plants (GS-5-3, GS-2-2, GS-2-3 and GS-2-4) were obtained by Basta® resistance screening. Transgene expression was confirmed by YFP fluorescence observation using a Zeiss LSM510 confocal fluorescence microscope.

### Generation and analysis of lines with dexamethasone-inducible RNAi

Dexamethasone-inducible constructs were made with the pOpOff2(hyg) destination vector system (Wielopolska *et al.*, 2005). The two targeted regions of the *SS4* gene are shown in Fig. S8, and oligonucleotide primers are in Table S1. PCR products SS4A and SS4B were cloned into the GATEWAY-ready pCR8/GW/TOPO TA entry plasmid and transferred into the destination vector to create pOpOff2(hyg)::SS4A and pOpOff2(hyg)::SS4B. These plus an empty vector were separately transformed into plants. Transformants were selected on media containing hygromycin, and single-copy homozygous lines were produced.

Ten-day-old plants were sprayed with 30 μM dexamethasone daily, 10 h into the 12-h light period, for 10 d. For measurements of *SS4* transcript and protein, plants were harvested immediately before spraying and 24 h after the final spraying. RNA was extracted using the RNeasyTM Plant Mini kit (Qiagen), digested with RQ1-DNAse (Promega), and used for cDNA synthesis. Semi-quantitative PCR was carried out with oligonucleotide primers listed in Table S1, using the *TUBULIN* gene as a control. For starch analysis, samples were taken at the end of the night and the day following the final spraying (14 h and 26 h afterwards, respectively).

### Microscopy

Sample preparation for electron and light microscopy was as described by Crumpton-Taylor *et al*. (2012) and Delatte *et al*. (2005).

### Starch analysis

Analysis of chain lengths was as described by Streb *et al*. (2008). Starch samples (100 mg) were boiled for 10 min in water, then debranched by incubation with isoamylase and pullulanase at pH 4.8 and 37°C for 2 h. Neutral glucans were separated by passage through sequential Dowex 50 and Dowex 1 minicolumns, lyophilized, redissolved, then subjected to HPAEC-PAD analysis on a Dionex PA-200 column. The relative proportions of each chain length in the total population (from d.p. 3 to d.p. 50) are expressed as a percentage of the total number of chains.

### Metabolite and enzyme assays

Starch, soluble glucans and sugars were extracted and assayed enzymatically (Critchley *et al.*, 2001; Delatte *et al.*, 2005). Other metabolites were measured by high pressure anion exchange chromatography coupled to tandem mass spectrometry (HPAEC-MS/MS: Lunn *et al.*, 2006). Leaves were frozen as rapidly as possible then extracted in chloroform/methanol. Where young and older leaves were harvested separately, rosettes were placed immediately after excision on a metal plate cooled to −80°C and the centre of the rosette was excised with a 1-cm diameter cork borer.

Starch synthase activity was assayed according to Jenner *et al*. (1994). To visualise glycogen and starch synthase activities, leaves were extracted with 100 mM MOPS, pH 7.2, 1 mM EDTA, 1 mM DTT, 10% (v/v) glycerol (300 mg leaf ml^−1^). Extracts (32-μl) were loaded onto nondenaturing polyacrylamide gels containing 0.3% (w/v) glycogen. After incubation for 16 h at 20°C in 100 mM HEPES-NaOH, pH 7.5, 2 mM DTT, 10% (v/v) glycerol, 0.5 mM EDTA, 0.5 M Na-citrate, 2 mM ADPglucose, starch and glycogen synthase activities were revealed by iodine staining.

### Immunoblotting

An antiserum was raised commercially in rabbits using the synthetic peptide DIGHDDGKNLDNIT (present in SS4 but not other starch synthase isoforms). Antibodies were affinity-purified using this peptide. Leaf tissue was powdered in liquid nitrogen then extracted in Laemmli sample buffer containing SDS and 1% (v/v) protease inhibitor cocktail (Sigma, www.sigmaaldrich.com). Electrophoresis and immunoblotting were as described by Barratt *et al*. (2001).

## Results

### Immature leaves of *ss4* plants contain almost no starch

We extended the characterisation of *ss4* mutants, using the *ss4-1* mutant allele (Roldán *et al.*, 2007) and a further T-DNA insertion line, *ss4-3*, which lacks SS4 protein (Fig. S1a).

Examination of whole rosettes and mature leaves largely confirmed previous reports of the *ss4* phenotype (Roldán *et al.*, 2007). Compared with wild-type plants, *ss4* plants had slightly reduced soluble starch synthase activities, 40% less chlorophyll (Table S2), less degradation of starch during the night (measured as end-of-day minus end-of-night starch contents) and higher end-of-night starch contents (Fig. S1b), and slower growth rates under both long and short photoperiods (Fig. S1c).

**Figure 1:**
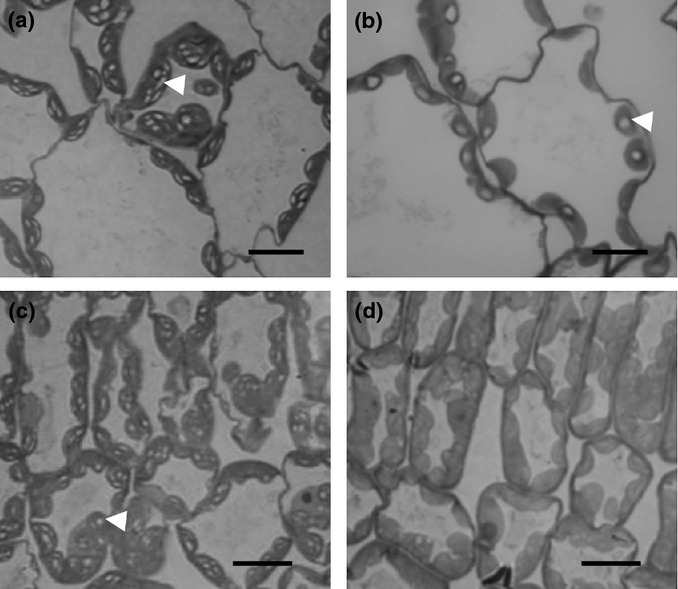
Light micrographs of sections of Arabidopsis leaves stained with toluidine blue. (a) Mature leaf, wild-type plant. (b) Mature leaf, *ss4* plant. (c) Immature leaf, wild-type plant. (d) Immature leaf, *ss4* plant (see also Fig.4a). (a–c) Arrows indicate starch granules. Bars, 10 μm.

As reported previously (Roldán *et al.*, 2007), mature *ss4* leaves appeared to have only one large, rounded starch granule per chloroplast (Figs 1a,b, S1d,e). Quantification of granule numbers (using Method 2 from Crumpton-Taylor *et al.*, 2012) revealed that mature, nonflowering rosettes of wild-type and *ss4-3* plants had 5.54 ± 0.28 and 0.87 ± 0.14 granules per chloroplast, respectively (mean ± SE from eight chloroplast preparations in both cases).

**Figure 2:**
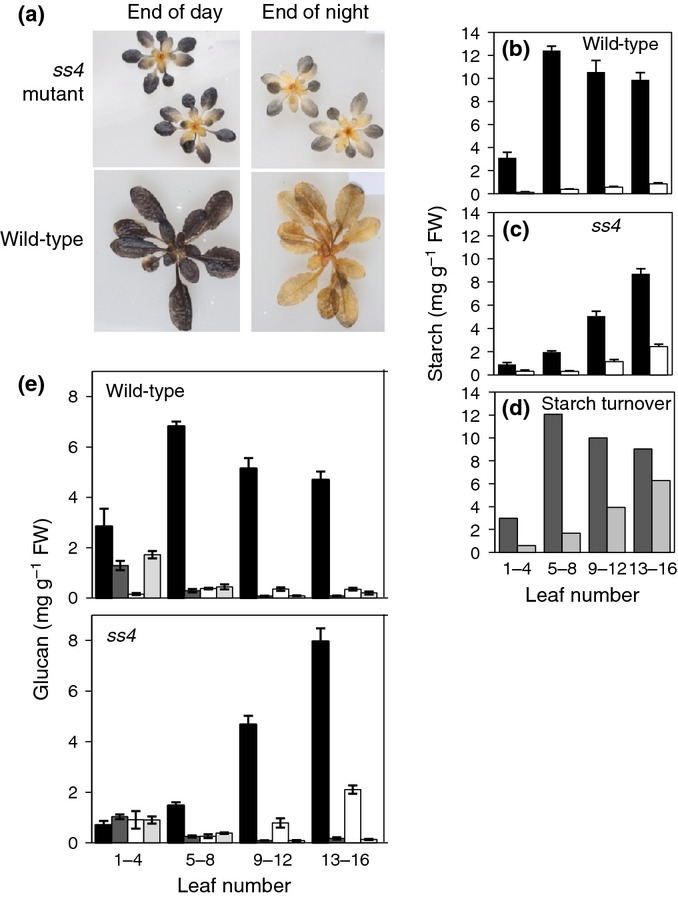
Starch and soluble glucan content and turnover in *ss4* leaves. (a) Presence of starch in rosettes of *ss4* and wild-type (Col) Arabidopsis plants. Decolourised rosettes were stained with iodine solution. (b) Starch contents of wild-type leaves at the end of the day (black) and the end of the night (white). Leaf 1 is the youngest and leaf 16 the oldest leaf. Values are means of measurements on four plants. Error bars, + SE. (c) Starch contents of *ss4* mutant leaves, as for (b). (d) Turnover (end-of-day minus end-of-night starch contents) in wild-type (dark grey) and *ss4* mutant (light grey) leaves, calculated from (b) and (c). (e) Starch and soluble glucan contents at the end of the day and the end of the night in leaves of wild-type and *ss4* mutant plants. Measurements were made on a separate batch of plants from those shown in (b–d). Leaf 1 is the youngest and leaf 16 the oldest leaf. Starch: black (end of day) and white (end of night). Soluble glucan: dark grey (end of day) and light grey (end of night). Values are means of measurements on at least five rosettes. Error bars, ± SE.

We found a different situation in immature leaves. Whereas immature leaves of wild-type plants have more starch granules per chloroplast than mature leaves (Crumpton-Taylor *et al.*, 2012), no starch granules were visible by light or electron microscopy in immature leaves of *ss4* plants (Fig.1c,d). Consistent with this observation, the youngest leaves of *ss4* rosettes did not stain with iodine at the end of the day (Fig.2a), and quantitative measurements revealed very low starch contents and little diel starch turnover (Fig.2c,d). Starch content and turnover increased with leaf age, but turnover was limited even in mature leaves. By contrast, in wild-type plants starch content and starch turnover were at their maximum in young leaves (leaves 5–8), and values were similar or somewhat lower in mature leaves (Fig.2b,d). Starch was not replaced with soluble glucan in *ss4* leaves. For both mature and immature leaves, *ss4* soluble glucan contents were lower than or comparable with wild-type contents (Fig.2e). Thus, loss of SS4 dramatically reduces the rate of acquisition of glucan storage and turnover capacity during leaf development. Mutants fail to form starch granules until late in leaf development, and then only about one granule is formed per chloroplast.

We examined whether loss of SS4 affected starch content in the primary root cap, a region of high starch content in wild-type plants. Starch in the columella cells is essential for the normal gravitropic response of the root (Kiss *et al.*, 1989; Blancaflor *et al.*, 1998; Kiss & Edelmann, 1999). There was wide variation in the amount and location of starch in root caps of *ss4* seedlings grown on vertical agar plates. Some *ss4* root caps were not distinguishable from those of wild-type plants, but in most cases starch content was reduced with some or all cells having no visible starch. Roots of *ss4* seedlings tended to deviate from vertical growth, and the degree of deviation was broadly negatively correlated with starch content (Fig. S2).

### Starch synthesis in immature *ss4* leaves is not restored by blocking starch degradation

We considered the possibility that starch granules are formed in immature *ss4* leaves, but are immediately degraded. To examine this possibility, we introduced into the *ss4* background a mutation that blocks starch degradation in wild-type plants. The *sex1* mutation affects glucan, water dikinase (GWD), a starch-phosphorylating enzyme that renders the surface of the starch granule accessible to starch degrading enzymes at night (Stitt & Zeeman, 2012). Mutants lacking GWD have a strongly reduced rate of starch degradation at night, and accumulate very high concentrations of starch in all leaves (Zeeman & ap Rees, 1999; Yu *et al.*, 2001).

**Figure 3:**
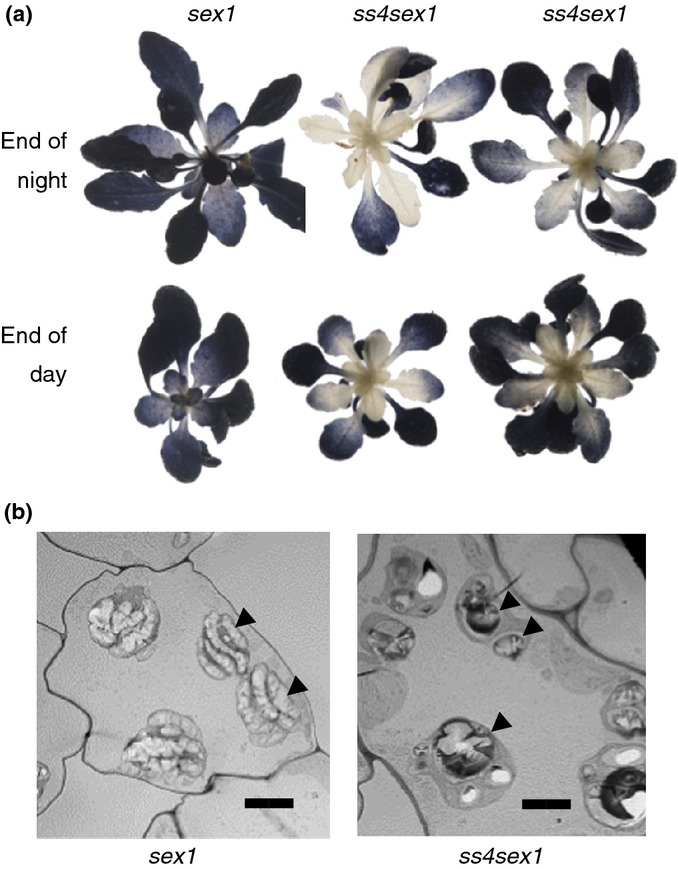
Effects of the *ss4* mutation in a *sex1* background. (a) Presence of starch in rosettes of *sex1*, and two independently selected *ss4sex1* double mutant Arabidopsis lines. Decolourised rosettes were stained with iodine solution. (b) Transmission electron micrographs of (left) a cell in a mature *sex1* leaf and (right) part of a cell in a mature *ss4sex1* leaf. The arrows indicate starch granules. Note that *sex1* starch granules are flattened, whereas *ss4sex1* granules are rounded and of very variable sizes. Bars, 4 μm.

The *ss4sex1* double mutants grew more slowly than *ss4* mutants, at about the same rate as *sex1* mutants (Fig. S3). Mature leaves had high starch contents at the end of both the day and the night. However, immature leaves contained very little starch and most chloroplasts appeared to contain no granules (Figs3, S3). Some profiles of chloroplasts in sections of mature *ss4sex1* leaves also had no starch granules and others contained few, rounded granules of variable size, whereas *sex1* chloroplasts in mature leaves were packed with flattened granules (Fig.3). Thus, introduction of a mutation that drastically reduces starch degradation in a wild-type background had only minor effects on starch granule formation in *ss4* leaves.

### Leaves of *ss4* mutants accumulate very high concentrations of ADPglucose

The experiments described above show that neither starch granules nor soluble glucans accumulate in immature *ss4* leaves. This observation suggests that the actions of starch synthase isoforms other than SS4 may be dependent on SS4. To test this idea, we measured the impact of the loss of SS4 on concentrations of the starch synthase substrate ADPglucose. Concentrations were 50–200 times higher in *ss4-1* and *ss4-3* rosettes than in wild-type rosettes, and were elevated in both mature and immature leaves (Tables1, S3). This was a highly specific effect. Analysis of mutants lacking any of the other three isoforms of soluble starch synthase (the *ss1*,*ss2* and *ss3* mutants) revealed less than two-fold differences between their ADPglucose concentrations and those of wild-type plants (Table 1). In the *ss4* mutant there were no large alterations with respect to wild-type plants in concentrations of soluble glucans (as previously shown, Fig.2e) or other intermediates of starch and sucrose synthesis, glycolysis and the Calvin–Benson cycle, and the Krebs cycle (Table 1).

**Table 1:** Metabolite contents of Arabidopsis rosettes lacking starch synthase isoforms

Metabolite	Amount (nmol g^−1^ FW)					
	Wild-type Col	Wild-type Ws	*ss1* (Ws)	*ss2* (Col)	*ss3* (Col)	*ss4-3* (Col)
ADPglucose	2.14 ± 0.88	2.32 ± 1.10	4.02 ± 1.16	1.78 ± 0.53	2.36 ± 0.40	124.2 ± 25.8
UDPglucose	105 ± 11	104 ± 14	111 ± 15	103 ± 11	107 ± 10	109 ± 4
Trehalose 6-P	0.23 ± 0.02	0.22 ± 0.03	0.27 ± 0.02	0.25 ± 0.02	0.31 ± 0.04	0.38 ± 0.05
Glucose 6-P	151 ± 43	175 ± 19	212 ± 17	167 ± 15	204 ± 12	265 ± 21
Glucose 1-P	51.0 ± 2.7	50.2 ± 5.2	51.8 ± 4.1	51.9 ± 3.8	54.0 ± 3.3	61.0 ± 3.9
Fructose 6-P	44 ± 15	46 ± 12	59 ± 8	48 ± 6	56 ± 7	69 ± 4
Fructose 1,6-bisphosphate	39 ± 6	35 ± 11	42 ± 7	41 ± 9	47 ± 7	59 ± 10
Glycerol 3-P	10.9 ± 1.0	9.7 ± 0.6	10.7 ± 1.4	10.7 ± 1.2	10.7 ± 1.3	8.5 ± 0.8
3-Phospho-glycerate	395 ± 86	407 ± 43	404 ± 60	334 ± 78	416 ± 55	386 ± 83
Phosphoenol-pyruvate	29 ± 6	27 ± 4	33 ± 8	26 ± 7	31 ± 8	34 ± 7
Pyruvate	101 ± 7	111 ± 10	122 ± 9	112 ± 9	92 ± 7	75 ± 10
Malate	1207 ± 671	1004 ± 271	1684 ± 441	1293 ± 234	1352 ± 267	1015 ± 250
Fumarate	1734 ± 616	1826 ± 357	2329 ± 289	2000 ± 223	1948 ± 365	1680 ± 218
Aconitate	91 ± 13	90 ± 8	95 ± 8	87 ± 9	93 ± 11	83 ± 6
2-Oxo-glutarate	84 ± 30	85 ± 13	105 ± 14	120 ± 10	102 ± 6	64 ± 6
Citrate	6103 ± 2438	5594 ± 1429	6441 ± 503	5957 ± 562	6299 ± 700	5710 ± 323
Succinate	92 ± 71	47 ± 42	143 ± 41	97 ± 39	100 ± 53	104 ± 45
Shikimate	21 ± 2	21 ± 3	21 ± 1	23 ± 2	22 ± 1	32 ± 3

Plants were grown together in identical conditions. Rosettes were excised *c*. 4 h into the 12 h photoperiod, and very rapidly frozen in liquid nitrogen. Metabolites were extracted in chloroform/methanol and assayed by anion exchange chromatography linked to tandem mass spectrometry. Values are means of measurements on six rosettes ± SD.

As expected of any mutant with a reduced capacity to generate sugars from starch at night (e.g. *pgm1*, Gibon *et al.*, 2004; *mex1*, Niittylä *et al.*, 2004; *lsf1*, Comparot-Moss *et al.*, 2010), sucrose concentrations were elevated in *ss4* plants during the day, and sucrose and hexose concentrations were lower in *ss4* than in wild-type plants during most of the night (Fig. S1b; see also Roldán *et al.*, 2007).

### Starch synthesis in an *ss4* background is partially but not fully restored by expression of *Agrobacterium* glycogen synthase

**Figure 4:**
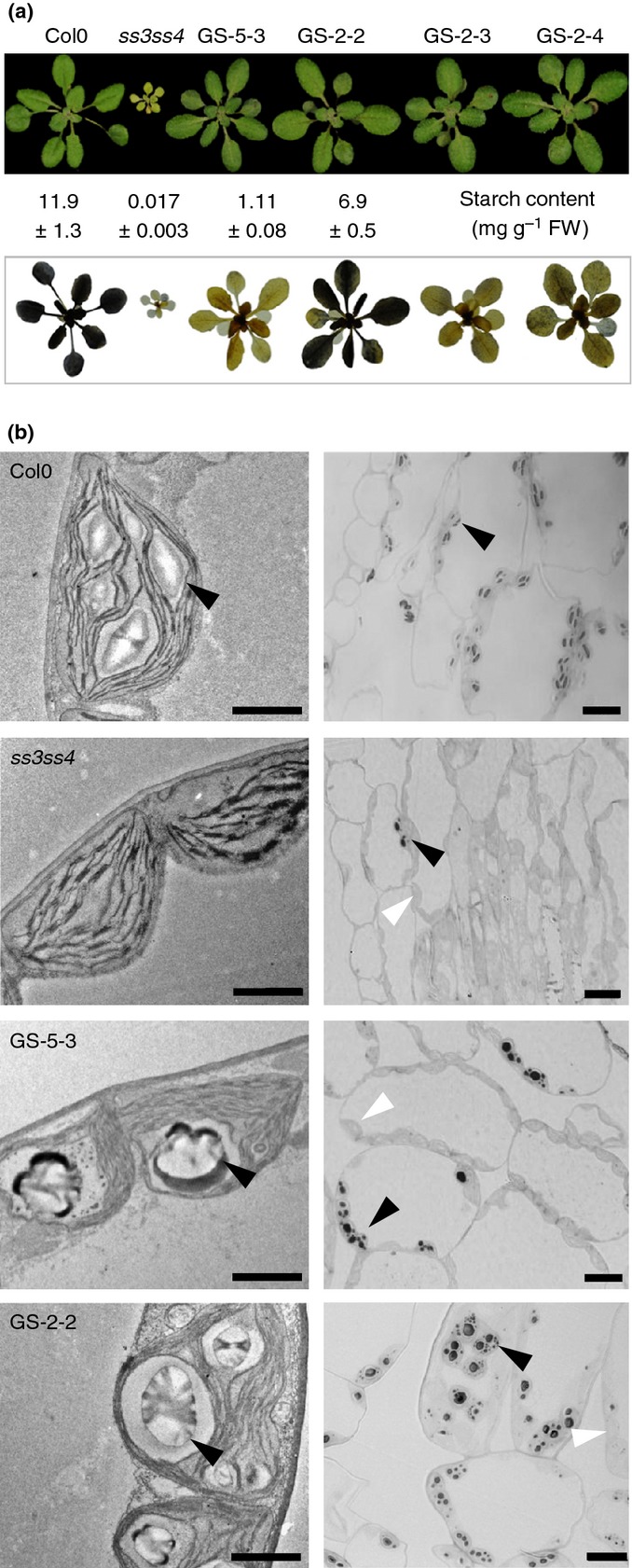
Expression of *Agrobacterium tumefaciens* glycogen synthase (GS) in the *ss3ss4* mutant of Arabidopsis. (a) A Col-0 wild-type plant, a *ss3ss4* mutant plant, and the T_2_ generation of four independent GS transformed *ss3ss4* lines. Upper panel, plants grown for 3 wk in 12-h light, 12-h dark conditions. Lower panel, plants harvested at the end of the day, decolourised and stained with iodine solution. Starch contents at the end of the day are given for wild-type, *ss3ss4* and two GS-transformed lines. Values are means ± SE of measurements on five 30-d-old plants. (b) Electron (left) and light (right) micrographs of mature leaves from Col-0, *ss3ss4* and GS-transformed *ss3ss4* plants, 10 h into the light period. Sections for light microscopy were stained with toluidine blue. Bars: 2 μm (for electron micrographs), 10 μm (for light micrographs). Black arrowheads indicate starch granules in plastids. White arrowheads indicate plastids that are devoid of starch.

The results above imply that SS4 directly or indirectly provides a ‘primer’ required for the actions of other starch synthases. To investigate this possibility, we overexpressed glycogen synthase (GS) from *A. tumefaciens* in an *ss4* background. We chose this enzyme because it is reported both to elongate glucan chains and to initiate chains *de novo* via an autoglucosylation mechanism (Ugalde *et al.*, 2003), using ADPglucose as its substrate. Thus it might replace the function of SS4 by providing glucosylated proteins as primers for other starch synthases, resulting in the formation of starch granules. We introduced the GS into plants lacking both SS4 and SS3 (the *ss3ss4* double mutant), because these mutants contain even fewer starch granules than the *ss4* mutant (Szydlowski *et al.*, 2009; Mérida & D′Hulst, 2012). Indeed, in our growth conditions, (12 h : 12 h, light : dark), *ss3ss4* plants were pale, slow growing and contained very little starch (Figs 4, S4).

*Agrobacterium* GS was introduced into *ss3ss4* plants as a fusion protein consisting of an N-terminal Rubisco small subunit chloroplast transit peptide, then a yellow fluorescent protein (YFP), then GS (Fig. S4). Examination of four independent GS-transformed *ss3ss4* plants with confocal fluorescence microscopy confirmed that the protein was targeted to chloroplasts. Analysis of crude extracts of leaves by nondenaturing PAGE on gels containing glycogen revealed abundant GS activity in these plants (Fig. S4).

All GS-expressing lines had higher growth rates and more starch than *ss3ss4* mutants (Figs4, S4). However, complementation was quantitatively and qualitatively incomplete. Although ADPglucose was reduced from 423 ± 113 nmol g^−1^ FW in *ss3ss4* plants to 40 ± 19 nmol g^−1^ FW in transformed line G-5-3 (see Figs4, S4), this concentration was still many times greater than that of wild-type plants grown under the same conditions (1.29 ± 0.23 nmol g^−1^ FW: means ± SD of measurements on three to five plants per line). Importantly, both the distribution and the nature of starch granules in the GS-expressing plants were very different from those of wild-type plants. In leaf sections, starch granules were visible in some chloroplasts but not in others. Furthermore, chloroplasts of a single cell appeared to contain a huge range of granule morphologies and sizes (Fig.4). As expected of amylopectin, polymers from starch granules in GS-expressing lines had a polymodal distribution of chain lengths. However, the pattern of distribution was not the same as that of amylopectin from wild-type leaves (Fig. S4). Thus, either the additional glucan-synthesizing capacity or the autoglucosylation activity provided by *Agrobacterium* GS can promote starch granule formation in the *ss3ss4* background, but neither of these functions of GS can fully restore normal starch synthesis.

### Starch synthesis in immature *ss4* leaves is partially restored by increased chloroplast volumes

The results above show that formation of new granules occurs much less frequently during leaf development in *ss4* leaves than in wild-type leaves. We showed previously that in wild-type plants the number of starch granules present in a chloroplast is a function of its volume, and that the number per unit volume is relatively constant for a given leaf developmental stage (Crumpton-Taylor *et al.*, 2012). Based on these observations, we speculated that the unit volume required per granule formation event might be much larger in *ss4* than in wild-type leaves. Average chloroplast volumes double over the course of leaf expansion (Crumpton-Taylor *et al.*, 2012), thus the delayed formation of granules in *ss4* leaves might reflect that fact that chloroplast volumes are larger in mature than in immature leaves.

In order to test this idea, we examined the impact on granule formation in the *ss4* background of the introduction of mutations that dramatically increase chloroplast volumes. The *accumulation and replication of chloroplast* (*arc*) mutants have few, giant chloroplasts per mesophyll cell, but the same number of starch granules per unit chloroplast volume as wild-type plants (Crumpton-Taylor *et al.*, 2012). Chloroplast sizes and numbers in *arc3ss4*,*arc5ss4*,*arc6ss4* and *arc10ss4* double mutants were similar to those in the respective *arc* parent (between one (*arc6*) and 25 (*arc10*) chloroplasts per cell, compared with ∼100 chloroplasts per wild-type cell, not shown). Nondenaturing PAGE analysis revealed no differences in activities of starch synthase isoforms other than SS4 between the parental and double mutant lines (Fig. S5).

**Figure 5:**
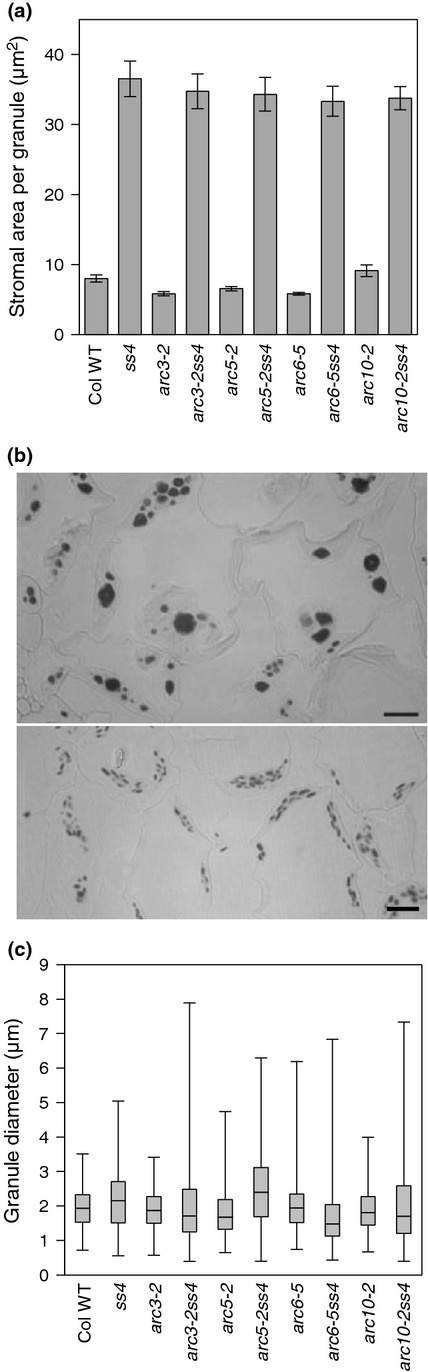
Effects of the *ss4* mutation in Arabidopsis *arc* mutant backgrounds. Values for single mutants are from Crumpton-Taylor *et al*. (2012). All genotypes were grown together in the same conditions. (a) Stromal area per starch granule for mature leaves of wild-type (WT), *ss4*,*arc* and *arc* x *ss4* mutants. Values are chloroplast cross-sectional area divided by numbers of granules in that area. Measurements were on either 40 chloroplasts (WT and single mutants) or 120 chloroplasts (*arc *× *ss4* mutants) in five random sections of two leaves from two different plants, harvested 9 h into the light period. Error bars, ± SE. (b) Sections of mature leaves, stained with iodine. Upper, *arc3-2ss4*; lower, *arc3-2*. Bars, 10 μm. (c) Granule diameters in wild-type, *ss4*,*arc* and *arc* x *ss4* plants. The solid line in the box is the median (50th percentile). The bottom and top of the box are the 25th and 75th percentiles. Upper and lower bars indicate the range of values. Between 319 and 2270 granules were measured on 11 randomly-selected scanning electron micrographs of starch from 40 25-d-old plants per genotype, at the end of the light period.

The double mutants differed from the *ss4* parent in that starch granules were visible in some chloroplasts in sections of immature leaves (Fig. S5) and starch turnover was comparable with that of the *arc* parent and wild-type plants (Fig. S5 h,i). However, starch synthesis in double mutants was different in several respects from that of *arc* and wild-type plants. Although some chloroplasts in immature *ss4* leaves contained granules, others apparently contained none (Fig. S5f,g). Granule numbers per unit chloroplast volume appeared to be lower than in wild-type plants throughout leaf expansion. In both immature and mature leaves, the range of granule sizes was larger in double mutants than in *ss4* or *arc* mutants, or wild-type plants (Figs5c, S5). The granules of double mutants were rounded rather than flattened, similar to those of *ss4* mutants (Fig S5). Thus, although granule formation in the absence of SS4 started earlier during leaf expansion development in *arc* mutant backgrounds than in a wild-type background, it was highly sporadic and qualitatively different from that in wild-type plants.

### Loss of SS4 from established plants affects granule numbers in immature but not mature leaves

Taken together, the observations above suggest that the primary effect of loss of SS4 is a strongly delayed and infrequent formation of new starch granules during leaf development. The alterations in granule number and starch turnover in mature leaves may be secondary consequences of this effect. To distinguish more clearly between primary and secondary effects, we reduced SS4 concentrations in mature rosettes by inducing expression of an RNAi hairpin construct targeted at the *SS4* gene, and examined the consequences for granule numbers and starch turnover.

**Figure 6:**
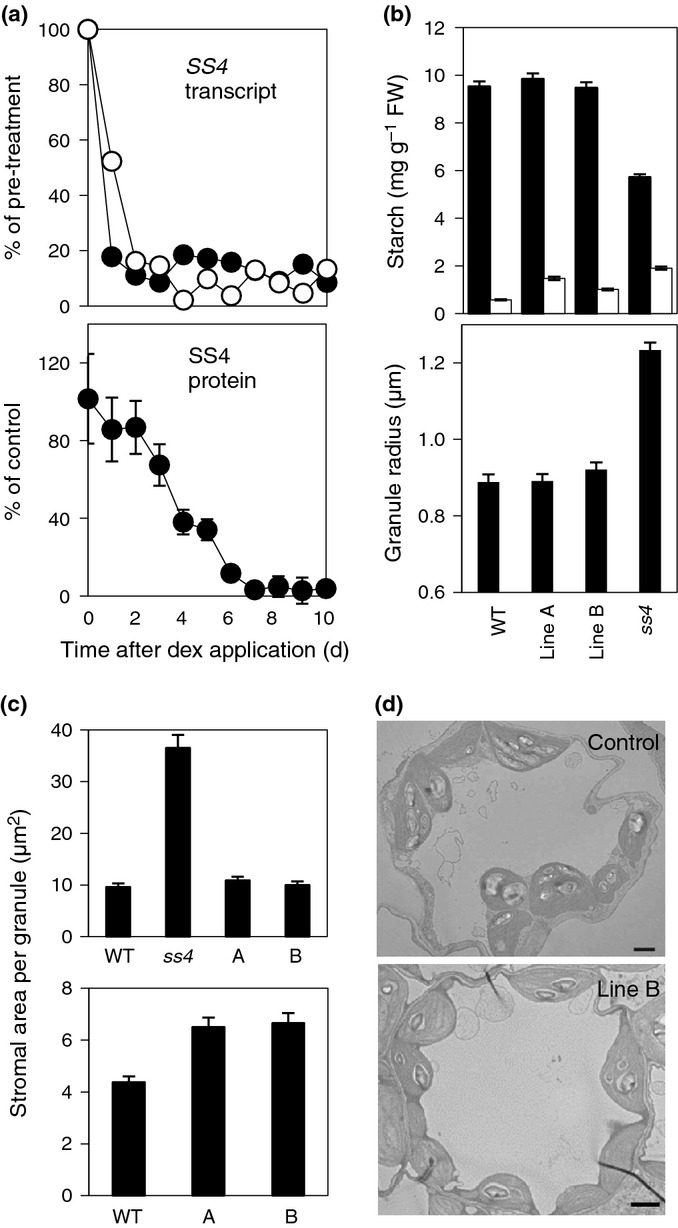
Effects of induction of expression of *SS4*RNAi in mature Arabidopsis rosettes. (a) Effect on *SS4* transcript and protein of daily application of dexamethasone (dex) for 10 d. Harvests were immediately before dex treatment, 10 h into the light period. For each harvest *SS4* transcript levels were expressed as a percentage of those of *TUBULIN*. These values are expressed as a percentage of the day 0 value. Closed circles, RNAi line A; open circles, RNAi line B. SS4 protein concentrations were estimated from immunoblots. Values are percentages of the day 0 value, and are means of measurements from six plants (three from line A, three from line B), all analysed on the same gel. Error bars, ± SE. Further details are in Fig. S6. (b) Starch content (upper panel) at the end of the day (closed bars) and the end of the night (open bars) and granule radii (lower panel) from wild-type, *ss4* and RNAi lines after 10 d of dex treatment. Starch values are means of measurements on eight rosettes. Radii of 250 granules per genotype were measured on starch from rosettes harvested at the end of the day. Error bars, ± SE. (c) Stromal area per granule for mature (upper) and immature (lower) leaves of wild-type (WT), *ss4* and RNAi line A and B plants after 10 d of dex treatment. Details are as for Fig.5(a) except that 40 chloroplasts were measured in seven sections. Harvest was 10 h into the light period. (d) Transmission electron micrographs of young leaves of wild-type and RNAi line B plants after 10 d of dex treatment. Bar, 2 μm.

Wild-type plants were transformed with constructs allowing expression of two hairpin RNAs based on different parts of the *SS4* gene sequence, under the control of a dexamethasone-inducible promoter (Figs S6, S7). Transgenic plants were treated with dexamethasone daily for 10 d. For each construct, one line showing strong reductions in SS4 protein was selected for further study. Before dexamethasone treatment, *SS4* transcript and protein concentrations were the same in transgenic lines and wild-type plants. *SS4* protein concentrations were unaffected by dexamethasone treatment in wild-type controls (Fig. S6b and not shown). In transgenic lines, *SS4* transcript levels declined to very low values within 24–48 h of the first dexamethasone treatment and remained low. Concentrations of SS4 protein fell slowly to undetectable levels over the first 7 d of dexamethasone treatment (Fig.6a).

After 10 d of dexamethasone treatment, whole rosettes of transgenic lines had higher end-of-night starch contents than control plants (although lower than in *ss4* plants), but diel starch turnover was at least 94% of that in wild-type plants and more than twice that in *ss4* plants. There was little or no difference between transgenic and control plants in whole-rosette starch content at the end of the day, average granule radius (Fig.6b) and granule size distribution (Fig. S6c). In sections of mature leaves, the chloroplast area per starch granule was the same in transformed and control plants. However, in immature leaves that had emerged during the dexamethasone treatment (leaves 1–4) the chloroplast area per starch granule was *c*. 50% greater in transgenic than in control plants (Fig.6c,d). In summary, reduced SS4 concentrations strongly reduced starch granule numbers in young leaves, but had little effect in mature leaves.

### SS4 concentrations can be substantially reduced without affecting starch granule formation

We investigated whether the amount of SS4 in a wild-type leaf limits starch granule formation. This possibility is suggested by a report that transgenic Arabidopsis plants with elevated concentrations of SS4 protein have increased rates of starch synthesis (Gámez-Arjona *et al.*, 2011). First, we examined starch turnover in leaves of *ss4* plants expressing the Arabidopsis *SS4* cDNA from the CaMV 35S promoter. Starch content and turnover were restored to near wild-type levels in both immature and mature leaves of several transgenic lines that had much lower concentrations of SS4 protein than wild-type plants (Fig. S8).

**Figure 7:**
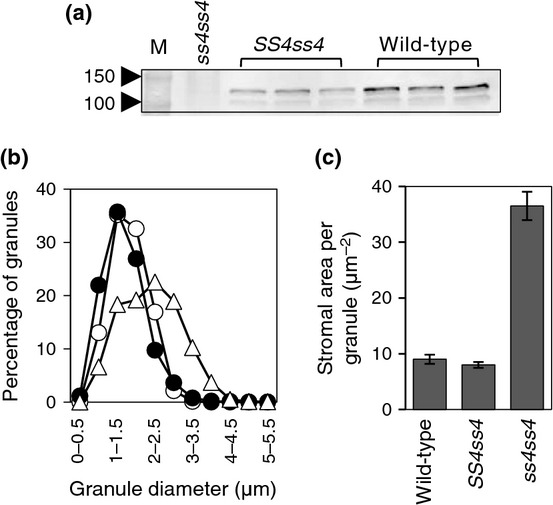
Starch characteristics of heterozygous (*SS4ss4*) Arabidopsis plants. (a) Immunoblot of extracts of mature wild-type, *ss4* mutant and *SS4ss4* rosettes, probed with SS4 antibodies (quantification: Fig. S9). M, molecular mass markers (in kDa). Each lane contains 40 μl extract; all extracts contained the same mg tissue per ml. (b) Diameters of granules extracted from wild-type (closed circles), heterozygote (open circles) and *ss4* (triangles) mature rosettes at the end of the light period. More than 500 granules were measured for each genotype. Each data point is the percentage of granules falling into the diameter range on the *x*-axis. (c) Stromal area per starch granule in mature leaves. Details are as for Fig.5(a) except that *c*. 50 rather than 40 chloroplasts were analysed. Wild-type and heterozygote values are not statistically significantly different.

Second, we examined growth and starch metabolism of heterozygous (*SS4ss4-3*) plants. As expected, both mature and immature leaves had approximately half the SS4 protein content of wild-type plants (Figs7, S9). Heterozygous plants were statistically significantly different from *ss4* mutant plants but not from wild-type plants with respect to FW, starch granule size distribution, granule volume, starch content and turnover in mature and immature leaves, and mean stromal area per starch granule (measured on light micrographs of leaf sections) (Figs7, S9). Thus, loss of half of the SS4 protein does not affect granule formation.

## Discussion

### SS4 is necessary for granule formation during leaf expansion

Our results show that SS4 is essential for the coordinated formation of starch granules that occurs during leaf expansion. In wild-type plants, chloroplasts have multiple starch granules from a very early stage of leaf development (Sakamoto *et al.*, 2009; Crumpton-Taylor *et al.*, 2012). Although chloroplasts undergo about three rounds of division as leaf cells expand, resulting in an eight-fold increase in chloroplast numbers (Marrison *et al.*, 1999), the numbers of starch granules per chloroplast remain relatively constant (a 1.5-fold decrease between early expansion and maturity: Crumpton-Taylor *et al.*, 2012). Thus, large numbers of new granules must be produced in association with rounds of chloroplast division. By contrast, no starch granules are visible in chloroplasts of immature leaves of *ss4* mutant plants. Granules appear only at a later stage of leaf expansion, and granule numbers per chloroplast are much lower in mature leaves than in wild-type plants.

These observations suggest that the low numbers of starch granules in chloroplasts of mature *ss4* leaves are a consequence of the major defect in granule formation during leaf expansion in this mutant. Support for this view comes from our analysis of plants in which concentrations of SS4 protein were progressively reduced by induction of RNAi specifically targeted at the *SS4* gene. Loss of SS4 protein was accompanied by reduced granule numbers in newly formed leaves but not in mature leaves, showing that SS4 is required for normal granule formation in immature leaves but not for maintenance of granule numbers in mature leaves. This conclusion is consistent with a specific role for SS4 in granule formation: although new granules arise frequently during leaf expansion this is likely to be a rare event once leaves reach maturity (Crumpton-Taylor *et al.*, 2012).

SS4 also appears to be necessary for a normal frequency of granule formation following cell division in root caps. The root caps of *ss4* plants generally contained less starch in fewer cells than wild-type plants, and there was a much higher degree of plant-to-plant variation in root cap starch content.

### SS4 plays a direct role in the formation of new granules

Loss of SS4 has several major effects on the Arabidopsis plant in addition to reduced starch granule numbers. These include reduced growth rates, altered patterns of starch turnover and concentrations of sugars, altered starch granule morphology, and reduced starch content and gravitropic response in roots. It is conceivable that failure of starch granule formation during leaf expansion is an indirect consequence of one or a combination of these effects. However, taking our results as a whole, we propose that SS4 is involved directly in the formation of granules, and that the other effects of its loss are secondary consequences of reduced granule numbers. The basis for this conclusion is as follows.

First, the absence of starch granules from immature leaves of SS4 mutants is likely to be caused directly by failure of granule formation rather than indirectly by alterations in other aspects of sugar or glucan metabolism. Immature *ss4* leaves did not accumulate abnormal concentrations of soluble glucans, but contained exceptionally high concentrations of ADPglucose, suggesting that in the absence of SS4 the remaining soluble starch synthases are largely inactive. The fact that introduction of the *sex1* mutation failed to restore either starch or glucan accumulation is also consistent with the idea that no starch is made. These observations point to a direct role for SS4 in a mechanism that generates a ‘primer’ that is acted on by other starch synthases, resulting in the formation of new granules.

Second, introduction of additional capacity for self-priming glucan synthesis in *ss4* leaves complemented some of the effects of loss of SS4 but did not restore normal starch synthesis. Expression of glycogen synthase in the highly compromised *ss4ss3* mutant reduced ADPglucose concentrations, increased starch concentrations, and restored wild-type appearance and growth rates. However, although starch granules were formed earlier in leaf expansion in plants expressing glycogen synthase, new granules arose sporadically and there were highly variable numbers per chloroplast. We conclude that glycogen synthase could catalyse the synthesis of glucans from which granules could be formed, but that it could not replace a specific and direct requirement for SS4 in a mechanism that controls the timing of granule formation and its coordination with chloroplast volume.

Third, in *ss4* mutants with increased chloroplast volume (*arc* × *ss4* mutants) some granules were present in immature leaves and starch turnover was comparable with that of wild-type plants, but granule distribution, shape and size remained highly variable. Thus, large chloroplast volumes permit more frequent formation of granules and higher starch turnover in the absence of SS4, but do not restore the coordinated production of typical transitory starch granules seen in wild-type leaves.

Fourth, comparison of the *ss4* phenotype with those of mutants lacking other components of the pathways of starch synthesis and degradation suggests that SS4 has an exclusive and direct role in granule formation. Several features of the *ss4* phenotype are also seen in other starch mutants. For example the starch synthesis mutant *pgm1* and the starch degradation mutant *sex1* – lacking plastidial phosphoglucomutase and glucan, water dikinase, respectively – have reduced growth rates, elevated daytime concentrations of sugars (Caspar *et al.*, 1985, 1991) and defective gravitropism (Caspar & Pickard, 1989; Vitha *et al.*, 2007). Other starch degradation mutants with reduced growth rates, reduced starch turnover and elevated daytime concentrations of sugars include *mex1*,*pwd1* and *dpe1*, lacking the chloroplast maltose transporter, phosphoglucan, water dikinase and the plastidial disproportionating enzyme, respectively (Critchley *et al.*, 2001; Niittylä *et al.*, 2004; Baunsgaard *et al.*, 2005; Kötting *et al.*, 2005). However, none of these mutants is reported to have reduced numbers of starch granules per chloroplast, or starch in mature but not immature leaves. Similarly, mutants lacking combinations of isoforms of starch synthase other than SS4 also exhibit reduced starch and elevated sugar contents and reduced growth rates, but are not reported to have reduced numbers of starch granules per chloroplast (*ss2ss3* mutants, Zhang *et al.*, 2008; *ss1ss2ss3* mutants, Szydlowski *et al.*, 2009). The impact of the *ss4* mutation on granule formation is thus likely to reflect a primary role for SS4 in this process, rather than a general secondary effect of perturbations in growth, starch and sugar metabolism, or gravitropism.

We suggest that most of the effects of loss of SS4 may be consequences of a primary failure of granule formation in immature leaves. As discussed above, the exceptionally high concentrations of ADPglucose may result from the absence of a primer required by other starch synthases. The amount of ADPglucose in *ss4* leaves is approximately the same as the total amount of ATP + ADP in wild-type leaves (*c*. 120 nmol g^−1^ FW: Strand *et al.*, 2000). The sequestration of most of the normal adenine nucleotide pool into ADPglucose probably compromises ATP regeneration during photosynthesis, and hence the operation of the Calvin–Benson cycle. The slow growth of *ss4* mutant plants may thus be due largely to reduced photosynthetic CO_2_ assimilation. Elevated daytime concentrations of sugars in *ss4* leaves are probably a secondary consequence of reduced starch turnover. The same phenomenon is seen in other mutants defective in starch turnover (as already described). The loss of gravitropism in roots of *ss4* mutants is highly likely to be a consequence of reduced formation of granules in the root cap. We observed a negative correlation between root cap starch content and deviation of the root from vertical growth. Reduced gravitropic responses have been observed previously in roots of Arabidopsis mutants with reduced starch contents (Caspar & Pickard, 1989).

Roldán *et al.* (2007) suggested that the low starch turnover in mature *ss4* leaves may result from the very small numbers of starch granules, which provide insufficient surface area for normal rates of synthesis and degradation. Our results provide some support for this idea. First, mature *ss4* leaves had elevated concentrations of ADPglucose, consistent with limitation of starch synthesis in these leaves by granule surface area. Second, the *arc* × *ss4* mutants, which had starch from an earlier stage of leaf expansion and a wider range of granule sizes than *ss4* mutants, also had higher daily starch turnover than *ss4* mutants. However, the possibility that SS4 is directly required for some aspect of starch turnover in mature leaves cannot be ruled out. SS4 can certainly participate in starch synthesis in mature leaves: leaves of triple mutant plants lacking the other isoforms of starch synthase (*ss1ss2ss3* mutants) are still capable of starch synthesis and turnover (Roldán *et al.*, 2007).

### SS4 is required for coordination of granule formation with chloroplast volume and for determination of granule morphology

*ss4* mutants are defective in the relationship between starch granule initiation and chloroplast volume, and in starch granule morphology. In wild-type plants, there is a tight relationship between stromal volume and numbers of starch granules. At a given stage of leaf expansion the number of granules per unit volume of stroma is remarkably constant, regardless of the total volume of the chloroplast. Granule sizes are also relatively uniform within and between chloroplasts (Crumpton-Taylor *et al.*, 2012). These relationships are absent in *ss4* mutants, and are not restored by manipulations that increase the frequency of granule initiation. Although immature leaves of *ss3ss4* mutant plants expressing glycogen synthase had more starch granules than those of *ss4* mutants, these granules were highly variable in distribution and apparent size. Whereas a few chloroplasts appeared to have large numbers of granules, many others had none. Transfer of the *ss4* mutation into an *arc* mutant background, in which chloroplast volumes are greatly increased, had a similar effect. Granule numbers in immature leaves of *arc* × *ss4* mutants were greater than in *ss4* mutants. However, granule distribution and size were highly variable, and there was no obvious relationship between starch granule number and chloroplast volume. We conclude that SS4 is an essential component of a mechanism that links granule initiation to chloroplast volume. In its absence, granule initiation appears to be stochastic.

Starch granules in leaves of *ss4* mutants are different in shape from those in wild-type plants. Rather than being flattened and discoid, they are rounded with an electron-transparent core (Roldán *et al.*, 2007;Fig. S1d). Manipulations of glucan-synthesizing capacity (by expression of *Agrobacterium* glucan synthase) and chloroplast volume (by introduction of *arc* mutations) did not restore normal granule morphology even though they increased the frequency of granule formation in immature leaves. Thus, the mechanism of granule formation in leaves in the presence of SS4 appears to be qualitatively different from that in its absence. It is interesting to note that flattened, discoid granules are found in leaves but not in other plant organs. Starch granules in nonphotosynthetic organs are generally rounded with a distinct core or hilum, resembling the leaf starch granules of *ss4* mutants. However, SS4 appears to be necessary for normal granule initiation in root caps as well as leaves, suggesting that it interacts with different granule-initiation mechanisms in the two organs.

What is the function of SS4 in granule initiation? As discussed above, it seems to be an essential part of a mechanism that generates a specific ‘primer’ on which other starch synthases can act, leading to granule formation. The primer might simply be small malto-oligosaccharides, perhaps elaborated by SS4 from ADPglucose alone or from maltose, which is produced *de novo* during photosynthesis (Linden *et al.*, 1975; Szecowka *et al.*, 2013). This explanation seems unlikely, however, because starch synthases other than SS4 are reported to synthesize malto-oligosaccharides from ADPglucose or maltose. Szydlowski *et al*. (2009) showed that recombinant SS3 can form glucans from ADPglucose alone. Recombinant SS1, SS2 and SS3 from kidney bean (*Phaseolus vulgaris*) can elongate both maltose and maltotriose (Senoura *et al.*, 2004, 2007). It is also difficult to explain why SS4 is required for normal granule shape, and why it cannot be fully replaced by *Agrobacterium* glycogen synthase, if its sole function is to provide small oligosaccharides on which other starch synthases can act. An alternative explanation (Szydlowski *et al.*, 2009; Mérida & D′Hulst, 2012) is that SS4 proteins adopt a quaternary structure – perhaps as part of a complex with other proteins – which serves as a synthetic and/or nucleation centre for specific glucans required for granule formation. This centre could also determine the subsequent direction of granule growth, and hence granule shape. Alone among the starch synthases, SS4 possesses coiled-coil domains in the noncatalytic n-terminal part of the protein. Coiled-coil domains can mediate protein–protein interactions (Mason & Arndt, 2004). Further work is required to establish precisely the nature of the primer made in the presence of SS4, and how it allows initiation of appropriate numbers of uniform granules of defined shape as chloroplasts expand and divide.

### SS4 is not limiting for starch granule formation in wild-type leaves

Although SS4 is required for the formation of normal transitory starch granules, it is unlikely that its concentration exerts significant control over the numbers of granules initiated in wild-type plants. First, granule numbers in *SS4ss4* heterozygotes with half the wild-type concentrations of SS4 protein were indistinguishable from those of wild-type plants. Second, normal starch metabolism was restored in *ss4* mutant plants by transgenic expression of SS4 at concentrations far below those in wild-type plants. These results imply that the tight relationship between stromal volume and numbers of starch granules is not simply a function of numbers of SS4 protein molecules, but is determined by a complex mechanism of which SS4 is an essential but nonlimiting component. In this light, it is interesting that overexpression of SS4 in wild-type Arabidopsis plants results in increased rates of leaf starch synthesis and up to 50% more starch at the end of the day (Gámez-Arjona *et al.*, 2011). Important new information about the role of SS4 may be gained from analysis of the total starch synthase activity and the size, number and distribution of starch granules in these overexpressing plants.

## Supporting Information

Fig S1 Characterization of *ss4* mutants. **Fig. S2** Growth of *ss4* roots. **Fig. S3** Phenotypes of *ss4sex1* mutants. **Fig. S4** Expression of *Agrobacterium glgA* in *ss3ss4* mutants. **Fig. S5** Starch granules in *arc* x *ss4* mutants. **Fig. S6** Effects of inducing RNAi targeted at the *SS4* gene. **Fig. S7** *SS4* coding sequence showing regions targeted by RNAi. **Fig. S8** Transgenic *ss4* plants expressing SS4. **Fig. S9** Further characterization of heterozygous (*SS4ss4*) plants. **Table S1 **Oligonucleotide primers used in this study **Table S2** Starch synthase activities and chlorophyll contents of *ss4* mutants **Table S3** ADPglucose contents of mature and immature leaves of *ss4* mutants
