# Supplementary material for: Starch synthase 4 is essential for coordination of starch granule formation with chloroplast division during Arabidopsis leaf expansion
Source: New Phytol. 2013 Aug 19;200(4):1064–75. doi: 10.1111/nph.12455 (PMC4283981; doi:10.1111/nph.12455)
Supplement: Fig S1 — Characterization of ss4 mutants. Fig. S2 Growth of ss4 roots. Fig. S3 Phenotypes of ss4sex1 mutants. Fig. S4 Expression of Agrobacterium glgA in ss3ss4 mutants. Fig. S5 Starch granules in arc x ss4 mutants. Fig. S6 Effects of inducing RNAi targeted at the SS4 gene. Fig. S7 SS4 coding sequence showing regions targeted by RNAi. Fig. S8 Transgenic ss4 plants expressing SS4. Fig. S9 Further characterization of heterozygous (SS4ss4) plants. Table S1 Oligonucleotide primers used in this study Table S2 Starch synthase activities and chlorophyll contents of ss4 mutants Table S3 ADPglucose contents of mature and immature leaves of ss4 mutants [file nph0200-1064-SD1.pdf]

## SUPPORTING INFORMATION

**Starch synthase 4 is essential for coordination of starch granule initiation with chloroplast division during Arabidopsis leaf expansion**

Matilda Crumpton-Taylor[1,*], Marilyn Pike[1,*], Kuan-Jen Lu[2], Christopher M. Hylton[1], Regina Feil[3], Simona Eicke[2], John E. Lunn[3], Samuel C. Zeeman[2] and Alison M. Smith[1]

[1]John Innes Centre, Norwich Research Park, Norwich NR4 7UH, UK; [2]Institute of Agricultural Sciences, Eidgenössische Hochschule Zürich, Universitätstr. 2, 8092 Zürich, Switzerland; [3]Max-Planck Institute of Molecular Plant Physiology, Wissenschaftspark Golm, Am Mühlenberg 1, 14476 Potsdam-Golm, Germany.

Author for correspondence: Alison M. Smith

Tel: +44 (0)1603 450622; Email: alison.smith@jic.ac.uk

Table S1.

Oligonucleotide primers used in this study.

Primers used in the selection of *arc* mutants were as described in Crumpton-Taylor *et al.* (2012). Gene-specific sequences for Gateway® primers are in bold.

| Experiment | Oligonucleotide | Sequence |
|---|---|---|
| *ss4* mutant complementation with *SS4* cDNA | SS4CDNA_F | ATGACGACGAAGCTATCGAGC |
| | SS4CDNA_R | CGTGCGATTAGGAACAGCTC |
| | GFP_pK7_F | CTGTTGTAGTTGTACTCCAGCTTGTGC |
| | SALK096130_R | GAATTTGCTTGGATGTTGAGGTTC |
| Selection of *ss4sex1* mutant | R1-4_F | CGGAAGTAAAATCTTTTACAG |
| | R1-17_R | AAGGATTATGGCGATGGAAAGGGT |
| SS4 RNAi: pOpOff2(hyg)::SS4A | SS4_P4_F | GGATTGGCACTGTTGAAGGT |
| | SS4_P4_R | CCGGACTGAAGAAGCAACTC |
| SS4 RNAi: pOpOff2(hyg)::SS4B | SS4_P6_F | TTGGAGCGAGCATTTAATCA |
| | SS4_P6_R | CTCACGTGCGATTAGGAACA |
| Expression of *glgA* in *ss3ss4* pDONR221:: *glgA* | glgA_F | GGGGACAAGTTTGTACAAAAAAGCAGGCTTG **ATGAATGTCCTTTCGGTTTCATC** |
| | glgA_R | GGGGACCACT TTGTACAAGA AAGCTGGGT**A TGGCCTTTCG AAATAAGCTG G** |
| *TUBULIN* as control in RNAi experiment | TUB_F | CCTGATAACTTCGTCTTTGG |
| | TUB_R | GTGAACTCCATCTCGTCCAT |

Table S2.

Starch synthase activities and chlorophyll contents of *ss4* mutants.

Measurements were made on mature, non-flowering rosettes. Starch synthase activity was measured on freshly prepared leaf extracts by a modification of the resin method of Jenner *et al*. (1994).

Values are means ± SE of measurements on five rosettes for each genotype.

| Genotype | Soluble starch synthase activity (µmol $min^{-1}$ $g^{-1}$ fresh weight) | Chlorophyll content (mg $g^{-1}$ fresh weight) | Chlorophyll a:chlorophyll b ratio |
|---|---|---|---|
| *ss4-1* | 0.128 ± 0.006 | 0.66 ±0.02 | 2.80 ± 0.03 |
| *ss4-3* | 0.140 ± 0.006 | 0.64 ±0.02 | 2.85 ± 0.08 |
| Wild-type | 0.181 ± 0.007 | 1.09 ± 0.06 | 2.84 ± 0.04 |

Table S3.

ADPglucose contents of mature and immature leaves of *ss4* mutants.

ADPglucose contents of two different *ss4* T-DNA insertion mutants, and of mature and young leaves from the same, mature, non-flowering rosettes. Values are means ± SD of measurements on five plants for each genotype for the batch of plants in the upper part of the Table, and for five plants per genotype for the separately-grown batch of plants in the lower part of the Table.

| Genotype/tissue | ADPglucose content (nmol $g^{-1}$ fresh weight) |
|---|---|
| *ss4-1* rosette | 42.4 ± 10.9 |
| *ss4-3* rosette | 43.6 ± 3.9 |
| Wild-type rosette | 0.58 ± 0.41 |
| *ss4-3*, immature leaves | 30.5 ± 11.2 |
| *ss4-3*, mature leaves | 26.2 ± 12.5 |
| Wild-type, immature leaves | 0.14 ± 0.10 |
| Wild-type, mature leaves | 0.13 ± 0.08 |

Figure S1

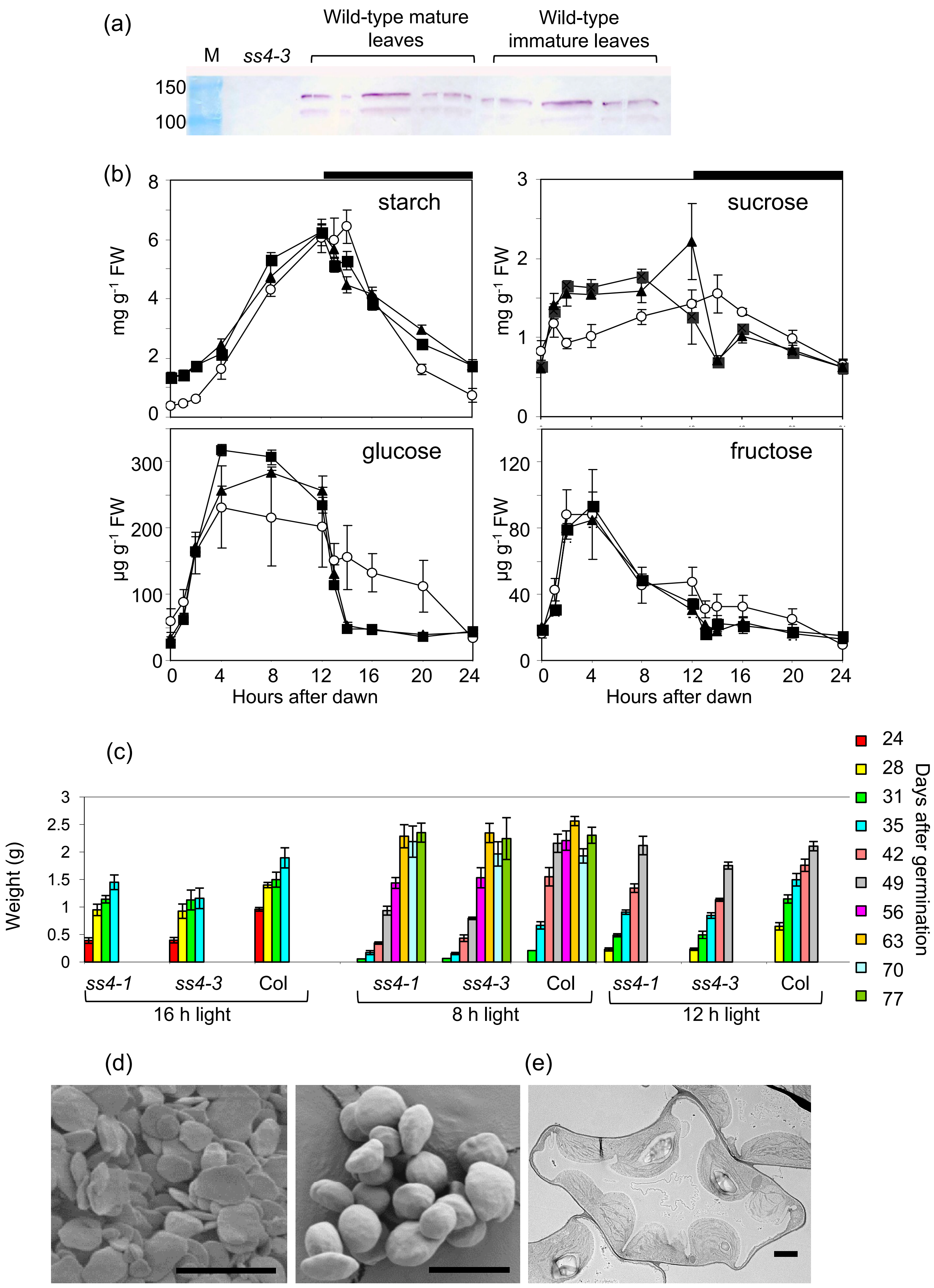

(a)
M
ss4-3
Wild-type mature leaves
Wild-type immature leaves
150
100
(b)
starch
sucrose
glucose
fructose
mg g-1 FW
µg g-1 FW
Hours after dawn
(c)
Weight (g)
ss4-1
ss4-3
Col
16 h light
8 h light
12 h light
Days after germination
24
28
31
35
42
49
56
63
70
77
(d)
(e)

**Fig. S1** Characterization of *ss4* mutants. (a) Immunoblot of an SDS-polyacrylamide gel of *ss4-3* mutant leaves and mature and young leaves of wild-type plants. M is molecular mass markers, masses indicated in kDa. Each of the remaining lanes contains 40 µl extract from a separate plant. All extracts contained the same mg tissue per ml extraction medium. Equal loadings were confirmed by SDS-PAGE followed by Coomassie InstantBlue$^{TM}$ staining of the same extracts (not shown). The blot was probed with purified SS4 antibodies, raised against a unique 14-amino-acid peptide. (b) Changes in starch, sucrose glucose and fructose over 24 h in mature, non-flowering rosettes grown under 12 h light, 12 h dark (darkness from 12 to 24 h after dawn). Values are means of measurements on six rosettes, bars are SE. Open circles, wild-type (Col). Filled triangles, *ss4-1*. Filled squares, *ss4-3*. (c) Fresh weights of rosettes of wild-type (Col) and *ss4* mutant plants grown in three different light regimes and harvested at the time points indicated. Light was 230, 148 and 136 µmol quanta $m^{-2}$ $s^{-1}$ in 16 h, 12 h and 8 h light periods respectively. In the 16-h light period all genotypes flowered at 24 d. In the 12-h light period Col flowered at 35 d and *ss4* mutants flowered at 42 d. In the 8-h light period Col flowered at 56 d and *ss4* mutants at 77 d. Data are means of measurements on five rosettes, error bars are SE. (d) Scanning electron micrographs of starch granules from wild-type (left) and *ss4* (right) rosettes harvested at the end of the light period. Bar represents 5 µm. (e) Transmission electron micrograph of a mesophyll cell in a mature leaf of a *ss4* rosette. Bar represents 2 µm.

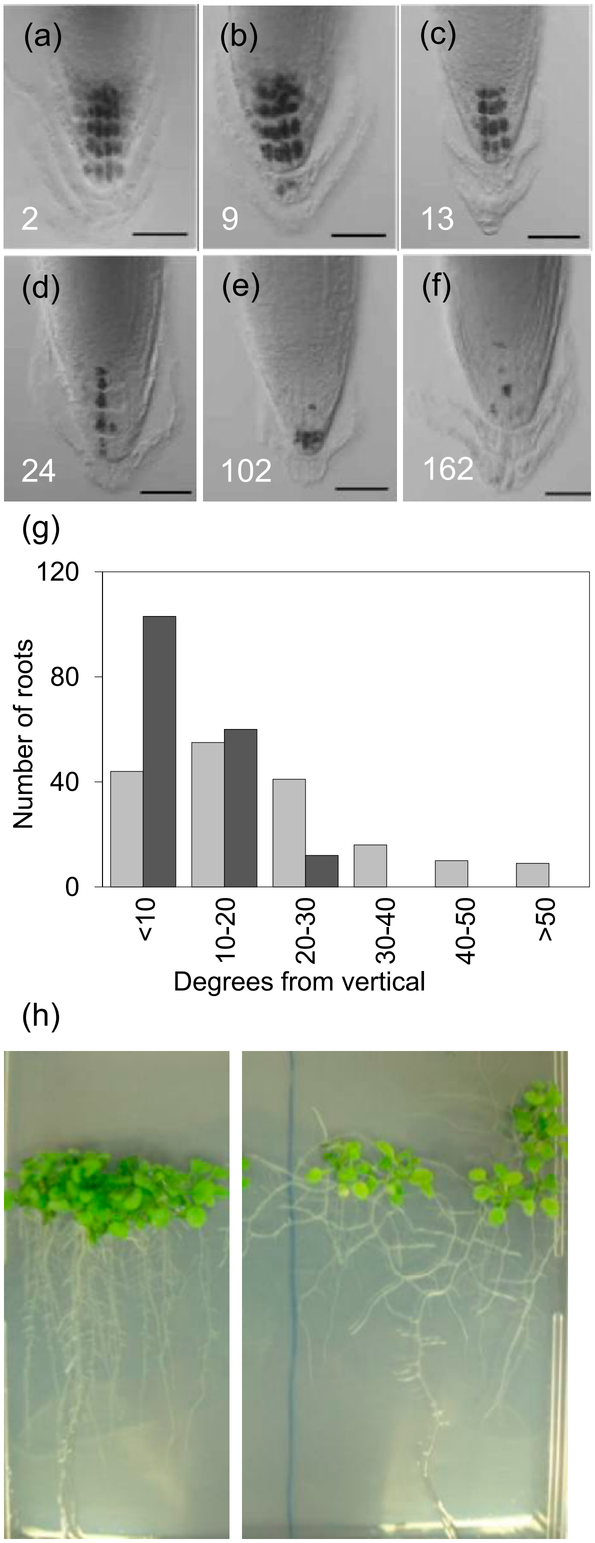


**Fig. S2** Growth of *ss4* roots. Seedlings were grown under sterile conditions in square polystyrene Petri dishes (100 x 15 mm) on 0.7% (w/v) agar containing the nutrients described in Haughn and Somerville (1986) with 1% (w/v) sucrose added. Seeds were surface-sterilised and sown on the agar medium in horizontal rows. After three days in darkness at 4 C, plates were placed vertically at 22 C under continuous illumination. (a) – (f) Iodine-stained root tips of wild-type (a) and *ss4* mutant (b) - (f) grown on vertical agar plates in 16 h light, 8 h dark, at four days (a), (c), (e), or eight days (b), (d), (f) after germination. Numbers indicate the deviation (degrees) of the apical 2.5 mm from the vertical. Bars represent 50 μm. (g) Deviation from the vertical of the apical 2.5 mm of wild-type (dark gray) and *ss4* (light gray) roots four days after germination. Measurements were on 175 plants of each genotype. (h) Seedlings of wild-type (left) and *ss4* mutant (right) plants eight days after germination on vertical agar plates.

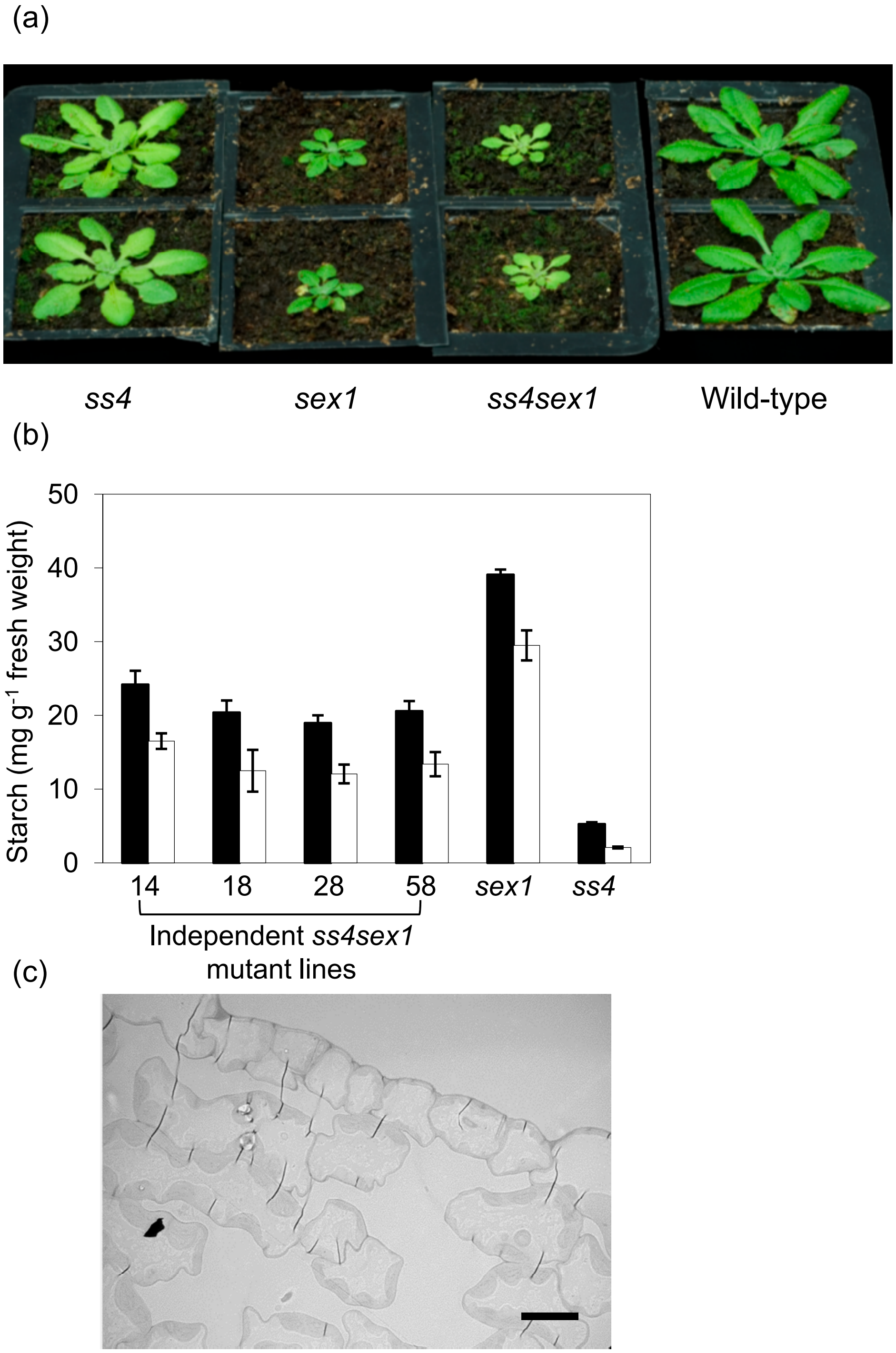


**Fig. S3** Phenotypes of *ss4sex1* mutants. (a) Appearance of wild-type, *ss4*, *sex1* and *ss4sex1* plants. Plants were of the same age and grown in the same conditions (12 h light, 12 h dark). (b) Starch contents of *sex1* and *ss4* plants and plants of four independently-selected *ss4sex1* mutant lines, at the end of the day (black) and the end of the night (white). Values are means of measurements on five or six rosettes. Error bars are SE. (c) Transmission electron micrograph of a young leaf of *ss4sex1*. Bar represents 10 µm.

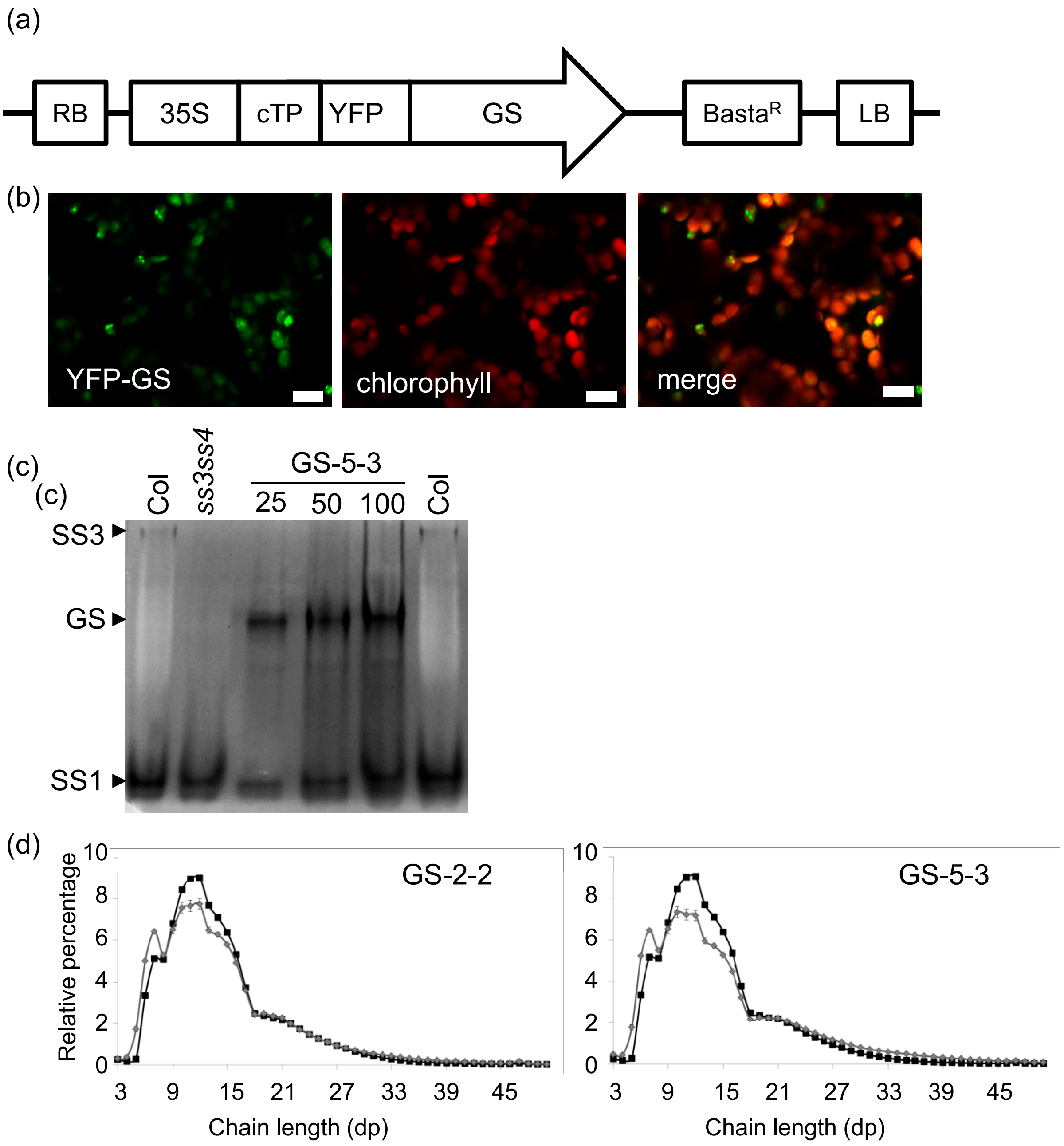


**Fig. S4** Expression of Agrobacterium *glgA* in *ss3ss4* mutants. (a) A construct for constitutive 35S-driven expression of GS fused to a chloroplast transit peptide (cTP and yellow fluorescent protein (YFP), was transformed into *ss3ss4* mutants. LB and RB: left and right T-DNA borders. Basta®: sequence encoding the BASTA resistant enzyme phosphinothricin acetyl transferase. (b) Localization of YFP-GS in transformed *ss3ss4* plants (line G-2-2). Confocal fluorescence micrographs showing YFP-GS (left), chlorophyll fluorescence (middle), and the merged images (right). (c) GS and endogenous starch synthase activities detected by non-denaturing PAGE. Soluble extracts of leaves were loaded onto native 7.5% polyacrylamide gels containing 0.3% (w/v) glycogen. For wild-type (Col) and *ss3ss4*, lanes contain material from 100 µg fresh weight. The three lanes for the GS-expressing line contain material from 25, 50 and 100 µg fresh weight. After electrophoresis and incubation in a medium containing 1 mM ADPG, activities were detected by iodine staining. GS and endogenous SS1 and SS3 activities are marked.

**Fig. S4** continued.

(d) Chain-length distribution of starch isolated from wild-type plants (black symbols) and s*s3ss4* lines expressing Agrobacterium GS (grey symbols). Left, GS-2-2. Right, GS-5-3. Starch was debranched with isoamylase and pullulanase and analysed by HPAEC-PAD. Peak areas were summed and the areas of individual peaks expressed as a percentage of the total. Values are means ± SE of measurements on four (wild-type, GS-5-3) or three (GS-2-2) independent samples.

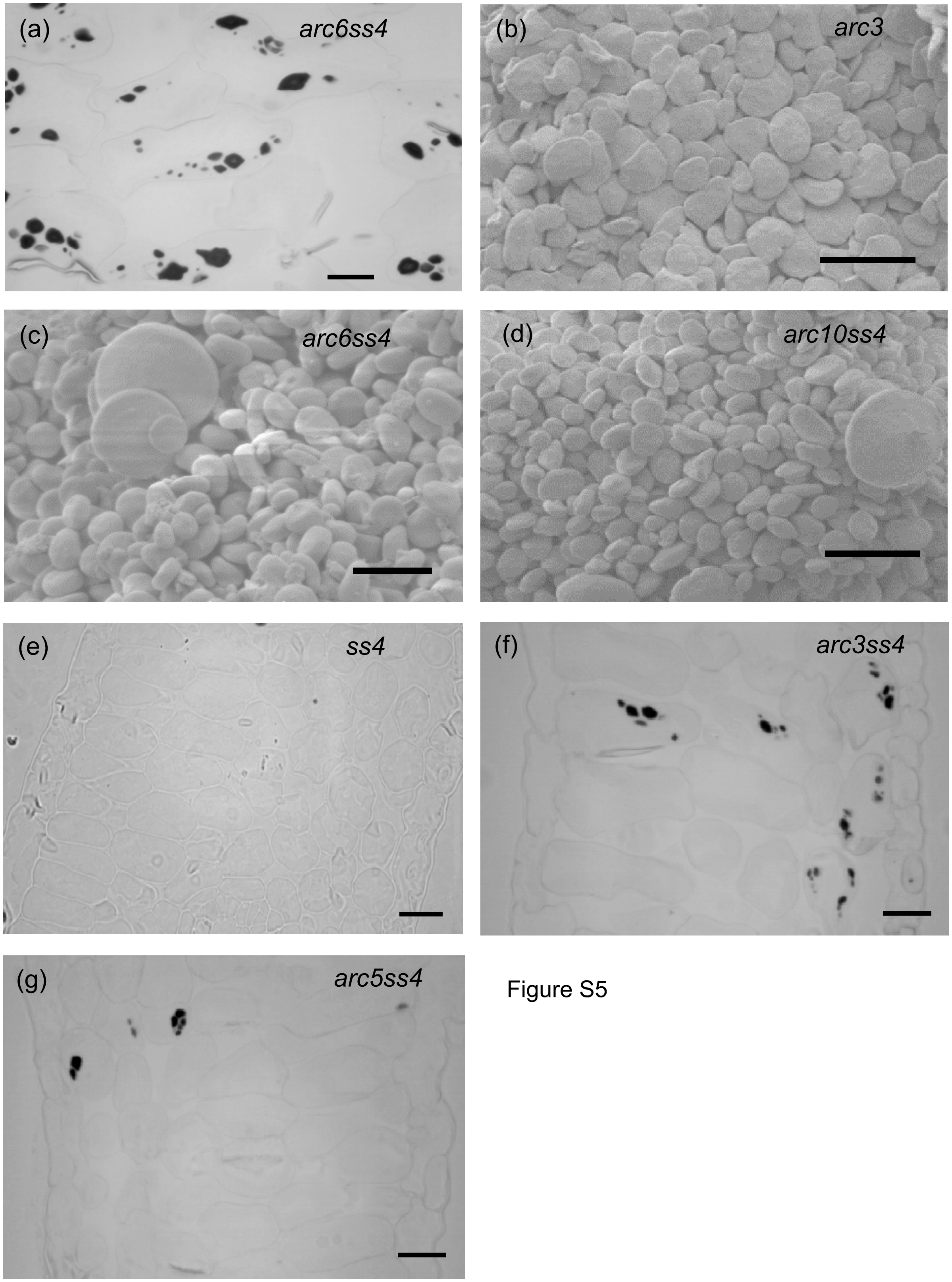
(a) arc6ss4
(b) arc3
(c) arc6ss4
(d) arc10ss4
(e) ss4
(f) arc3ss4
(g) arc5ss4

Figure S5

Figure S5 continued

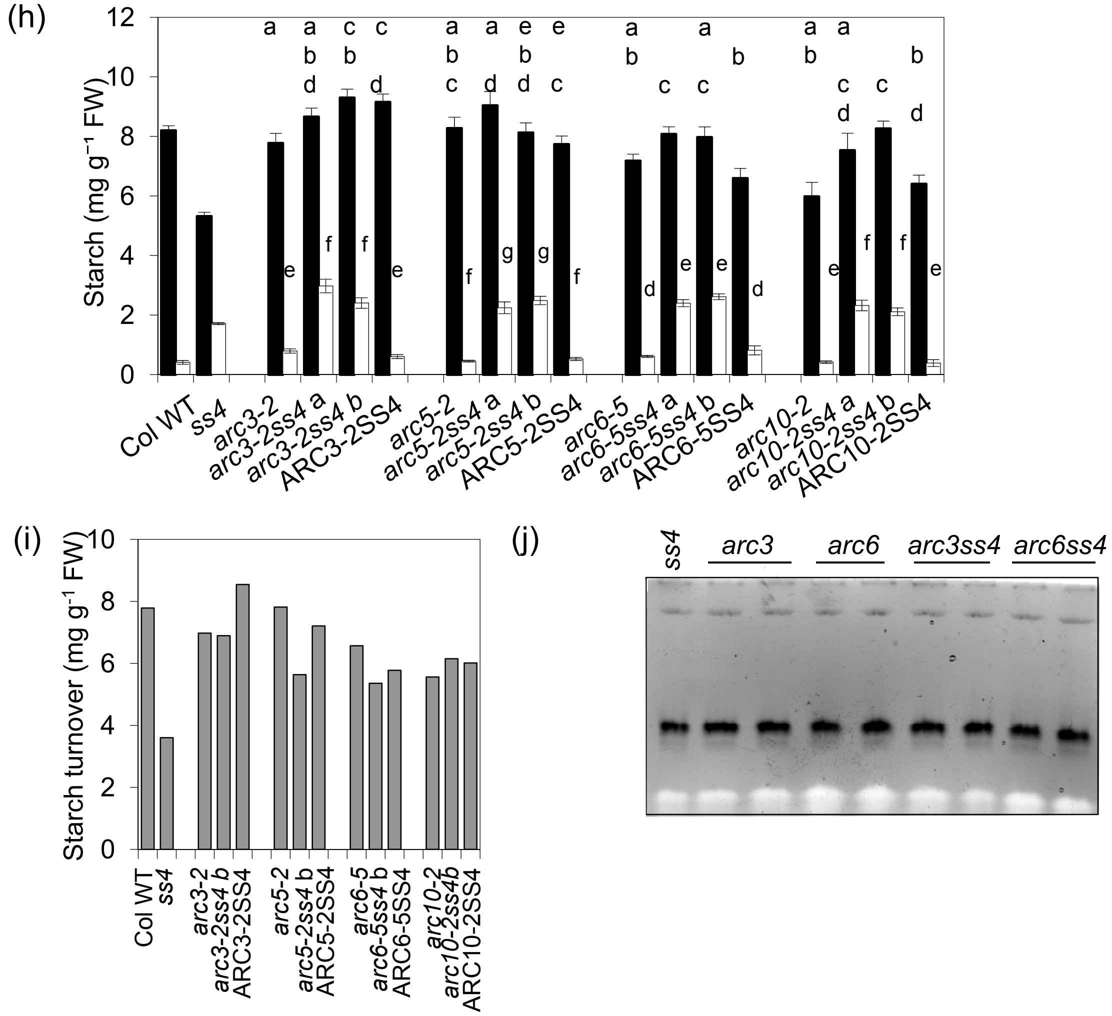


**Fig. S5** Starch granules and starch synthases in *arc* x *ss4* mutants. Light micrographs of leaf sections [(a), (e) – (g): iodine stained, bars represent10 µm] and scanning electron micrographs of starch granules from rosettes harvested at the end of the light period [(b) - (d): bars represent 5 µm]. Note that starch granules are present in some chloroplasts of the double mutants but not the *ss4* mutant, and that granules of double mutants are more rounded than those of *arc3*, with some exceptionally large granules in double mutants. (a) Mature *arc6ss4* leaf, stained with iodine solution. Bar represents 10 µm. (b) Starch granules of *arc3.* (c) Starch granules of *arc6ss4*. (d) Starch granules of *arc10ss4*. (e) Immature leaf of *ss4*. (f) Immature leaf of *arc3ss4*. (g) Immature leaf of *arc5ss4.* (h) Starch contents of leaves at the end of the day (black) and the end of the night (white) for wild-type, *ss4* and *arc* mutant plants, in *arc* x *ss4* mutants (two independently selected line for each genotype, designated a and b), and in out-segregating wild-type plants from *arc* x *ss4* crosses (e.g. *ARC3-2SS4*). Values are means of measurements on six to eight plants. Bars are SE. Values with the same letter are not statistically significantly different (p >0.05, Student's t-test). (i) Daily starch turnover in genotypes shown in (h). Turnover is end-of-day minus end-of-night starch contents, calculated from (h). (j) Starch synthase activities detected by non-denaturing PAGE. Soluble extracts of leaves (equivalent fresh weight in each lane) were loaded onto native 7.5% polyacrylamide gels containing 0.3% (w/v) glycogen. For all genotypes except *ss4*, the two lanes contain extracts from separate plants. After electrophoresis and incubation in a medium containing 1 mM ADPG, activities were detected by iodine staining. Note that band pattern and intensity is essentially the same in all genotypes.

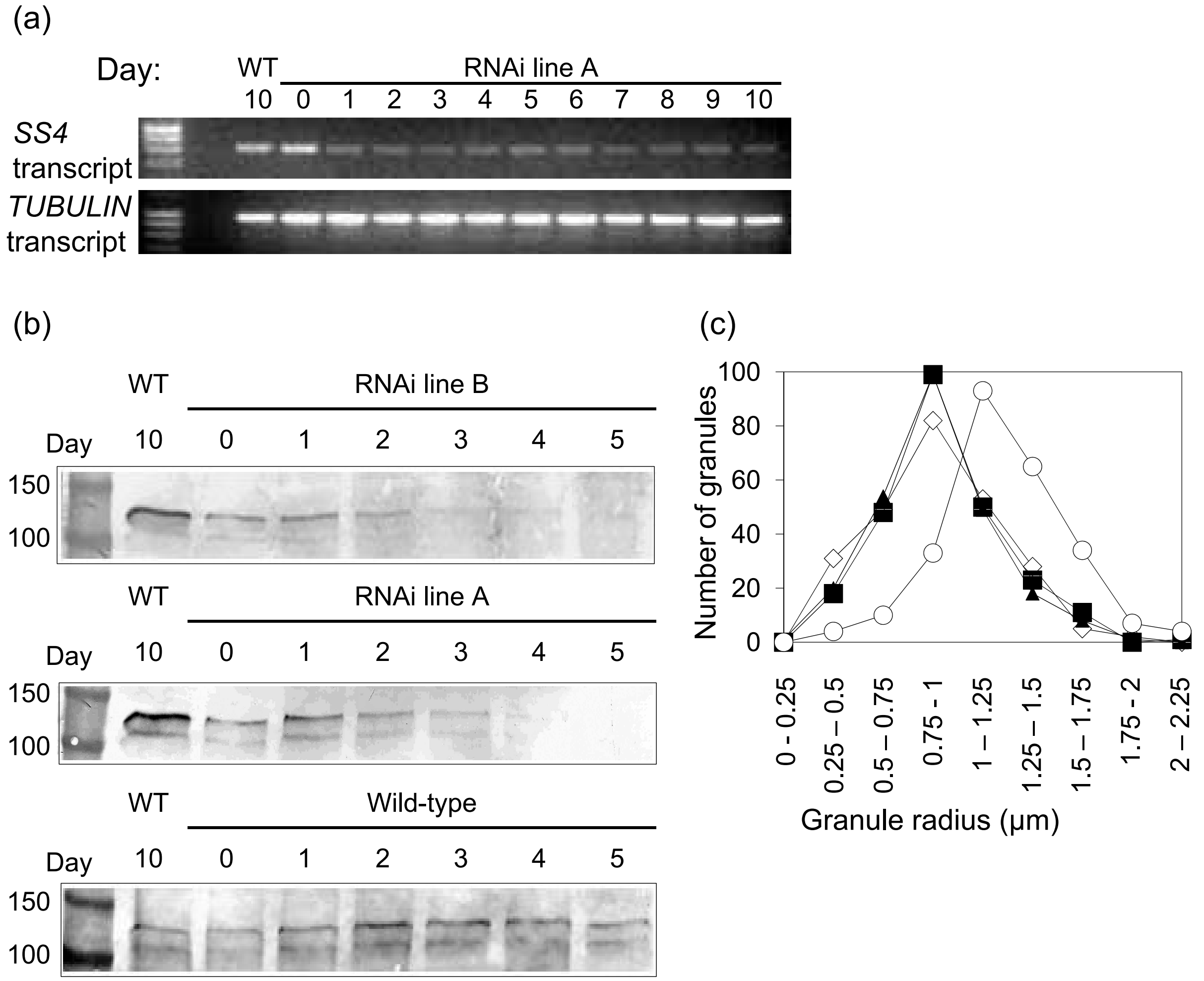


**Fig. S6** Effects of inducing RNAi targeted at the *SS4* gene. (a) Examples of agarose gels of PCR products for *SS4* and *TUBULIN* from RNAi line A , from which transcript levels shown in Figure 7(a) were derived. The first harvest (day 0) was immediately prior to dexamethasone (dex) application, 10 h into a 12-h light period. Dex was applied daily at this time point for the next ten days. Each harvest was immediately prior to dex treatment. Dex was also applied to wild-type (WT, not transformed) plants, which were harvested after 10 days of treatment. The left lane shows size markers of 1000, 800, 600 and 400 bp from top to bottom. (b) Representative immunoblots of SDS-polyacrylamide gels of extracts of wild-type, RNAi A and RNAi B plants, harvested at the indicated times after first application of dex. The positions of molecular mass markers are indicated at the right, in kDa. Each lane contains 40 μl extract from a separate plant. All extracts contained the same mg tissue per ml extraction medium. Experimental details are as in (a). Each harvest was immediately prior to dex treatment on days 1-5; wild-type (WT) plants were also harvested on day 10. Blots were probed with purified SS4 antibodies, raised against a unique 14-amino-acid peptide. These immunoblots are examples only. The protein levels displayed in Fig. 6(a) were derived from immunoblots of extracts of six separate plants per genotype for each time point. (c) Radii, measured from scanning electron micrographs, of starch granules extracted from wild-type (diamonds), RNAi line A (triangles), RNAi line B (squares) and *ss4* mutant (circles) rosettes at the end of the light period. Values are means of measurements on 250 granules for each genotype.

ATGACGACGAAGCTATCGAGCTTCTGTTTCTTAACCCATGGATTAGCAGGAATCTCCTGCGAGAGAGAACATGGAAGTTCTCGT
CGATTTTTCTATCTCCCTTCTCGTCGATTAGTTTCTACTTCGTGTAAGATGCGACAACAACGTGGTTTCGACTCTAGTAAAAGACA
AGAGGTCAAGAAAGGCTCCCCTAAACCGATCCTATCTATAAATTCAGGTCTTCAGAGCAACAATGATGAGGAATCTGATCTGGA
GAATGGTTCTGCGGACAGTGTCCCGAGTCTTAAGTCAGATGCTGAAAAGGGTAGTAGTATTCATGGCAGCATAGATATGAATCA
TGCTGATGAGAATCTTGAAAAGAAAGATGATATACAAACAACTGAAGTTACTCGGCGCAAGAGTAAAACTGCAAAGAAGAAAGG
GGAGAGTATTCATGCTACGATTGATATTGGACATGATGATGGGAAGAATTTAGATAATATTACTGTGCCTGAGGTTGCAAAAGCT
TTGTCCCTTAACAAAAGTGAAGGCGAGCAGATTTCAGATGGACAGTTTGGGGAACTAATGACAATGATAAGAAGTGCAGAAAAA
AATATTCTTCGGCTTGATGAAGCACGGGCCACTGCTCTTGACGACCTTAACAAGATTCTTAGTGATAAGGAAGCGTTGCAGGGA
GAAATCAATGTCTTGGAAATGAAATTGTCTGAGACTGATGAAAGGATTAAAACTGCTGCTCAAGAGAAAGCACATGTAGAGCTTC
TGGAAGAACAGTTAGAGAAGCTTCGTCATGAAATGATCTCCCCTATAGAAAGTGATGGTTATGTTCTAGCTCTTAGCAAAGAGCT
TGAAACATTGAAGCTGGAGAATCTATCTTTGAGAAACGATATAGAAATGCTTAAGTCAGAACTTGACAGTGTTAAAGATACCGGT
GAACGTGTGGTTGTGTTGGAAAAGGAGTGCTCCGGTTTGGAATCTTCTGTAAAGGATTTGGAATCTAAGTTATCGGTTTCTCAG
GAAGATGTCTCGCAGCTTTCTACTCTTAAAATCGAATGCACTGATCTATGGGCGAAGGTAGAGACTCTACAGCTGTTGTTAGATA
GAGCTACCAAACAAGCAGAGCAAGCAGTTATAGTGTTACAACAGAACCAAGATCTAAGAAATAAGGTCGACAAAATTGAGGAAT
CACTTAAAGAAGCCAACGTTTATAAAGAATCTTCAGAGAAAATTCAACAGTACAATGAGCTAATGCAACATAAGGTGACATTACT
TGAGGAGCGGCTCGAAAAGTCTGATGCAGAGATATTCTCATATGTTCAGTTATATCAAGAATCGATAAAAGAATTCCAGGAAACA
CTTGAAAGTTTGAAGGAAGAAAGCAAGAAAAAATCAAGAGATGAACCGGTTGATGATATGCCTTGGGATTATTGGAGTCGGTTA
CTTTTAACTGTTGATGGATGGCTGCTTGAAAAGAAAATAGCAAGCAACGATGCTGACTTACTGAGAGACATGGTATGGAAGAAA
GATCGAAGAATTCATGATACCTATATTGACGTCAAAGATAAGAATGAACGTGATGCCATCTCTGCATTTCTCAAGCTTGTCTCAT
CTCCAACAAGTTCAGGATTGTATGTCGTTCACATTGCAGCTGAGATGGCACCTGTAGCTAAGGTGGGAGGTTTGGGAGATGTTG
TGGCAGGTCTTGGTAAGGCATTGCAAAGAAAAGGTCATCTGGTGGAGATTATTCTTCCCAAATATGACTGTATGCAGTATGATC
GCGTGCGTGACTTAAGGGCTTTGGATACTGTTGTGGAGTCATATTTTGATGGGAAGTTATATAAAAACAAAATCT**[GGATTGGCA
CTGTTGAAGGTTTACCTGTACATTTCATTGAACCTCAACATCCAAGCAAATTCTTCTGGAGAGGACAGTTTTATGGAGAGCAA
GATGATTTCAGACGCTTCTCGTATTTTAGCCGAGCTGCACTAGAGTTGCTTCTTCAGTCCGG]**CAAAAAACCCGACATCATACAT
TGTCATGACTGGCAAACAGCTTTTGTTGCGCCGCTGTATTGGGATCTGTATGCTCCAAAGGGATTAGATTCTGCAAGAATATGC
TTTACATGTCATAATTTTGAGTATCAAGGTACCGCGTCTGCTTCAGAATTGGGATCTTGCGGGCTTGATGTTAACCAGTTAAATA
GACCAGACAGAATGCAGGATCACTCGTCTGGAGATAGAGTCAATCCTGTTAAGGGCGCTATAATTTTCTCAAACATTGTAACAA
CCGTGTCCCCTACTTATGCACAAGAAGTTCGAACAGCTGAGGGAGGAAAAGGACTCCATTCAACACTCAATTTTCACTCCAAGA
AATTCATTGGAATCCTCAACGGCATTGACACAGATTCATGGAATCCTGCCACCGACCCCTTCCTCAAGGCTCAGTTCAACGCTA
AAGATCTACAAGGGAAAGAAGAAAACAAACACGCCCTTAGAAAGCAGCTTGGACTTTCTTCAGCGGAGTCAAGACGGCCTTTG
GTTGGTTGCATAACAAGATTAGTACCACAGAAAGGAGTTCATCTAATCAGACATGCCATATACAGAACATTAGAGTTAGGTGGAC
AATTTGTACTTCTTGGTTCTAGCCCGGTTCCACATATTCAGAGGGAATTTGAAGGTATTGAACAACAGTTTAAAAGCCATGATCA
TGTCCGGTTGTTACTGAAGTACGATGAAGCTCTGTCTCATACGATTTACGCAGCCTCCGATTTGTTCATCATTCCGTCAATTTTT
GAGCCTTGTGGACTTACACAGATGATCGCTATGAGATATGGCTCCATTCCAATTGCGCGAAAAACTGGAGGCTTAAATGACAGT
GTCTTCGACATTGATGATGATACAATACCAACACAGTTTCAAAATGGATTTACATTTCAAACCGCGGACGAACAGGGTTTCAATT
ATGCG**[TTGGAGCGAGCATTTAATCACTACAAGAAAGATGAAGAGAAATGGATGAGACTTGTAGAGAAAGTGATGAGTATAG
ATTTCAGCTGGGGATCATCAGCTACACAATACGAAGAACTCTACACGAGATCAGTGTCCAGAGCAAGAGCTGTTCCTAATCG
CACGTGAG]**

**Fig. S7** *SS4* coding sequence showing regions targeted by RNAi. Primer regions are highlighted in red and the rest of the targeted sequence in blue. The first sequence is the target of the RNAi in line A; the second is the target of the RNA in line B (see Figs 6, S6).

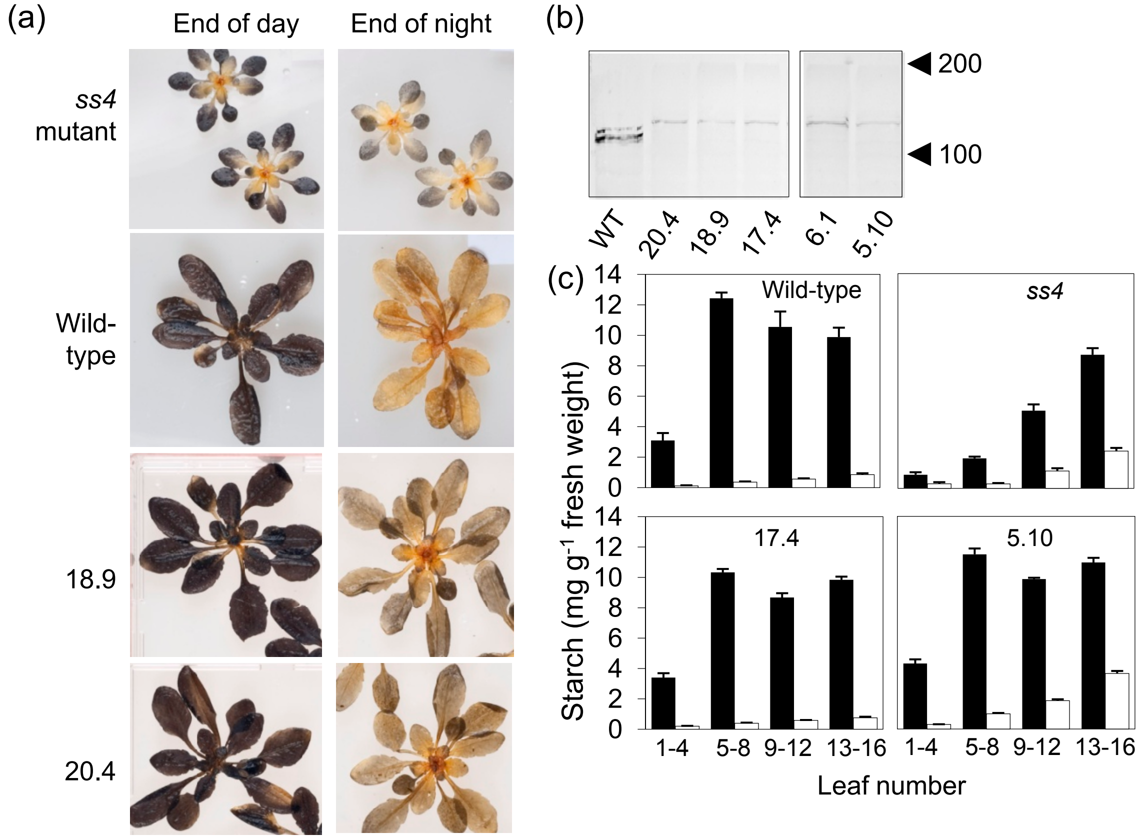


**Fig. S8** Transgenic *ss4* plants expressing SS4. (a) Starch contents of leaves of two *ss4* mutant lines expressing GFP-tagged SS4, at the end of the day (black) and the end of the night (white). Leaf one is the youngest and leaf 16 the oldest leaf. Values are means of measurements on four plants. Bars are SE. Note that the pattern of leaf starch content with respect to leaf age is similar to that of wild-type plants rather than *ss4* plants (compare with Fig. 2b, c). (b) Immunoblot of an SDS-polyacrylamide gel of extracts of rosettes of a wild-type plant and five lines of *ss4* plants expressing GFP-tagged SS4. The positions of molecular mass markers are indicated at the right, in kDa. Each lane contains 60 µl extract from a separate plant. All lanes are from the same gel, and blots were developed together and for the same length of time. All extracts contained the same mg tissue per ml extraction medium. The blot was probed with purified SS4 antibodies, raised against a unique 14-amino-acid peptide. SS4 appears as two bands in wild-type extracts (see Fig. S1a and Roldán *et al*., 2007). In the transgenic lines SS4 has reduced mobility because of its GFP tag (~30 kDa). No band is present at this position in immunoblots of extracts of *ss4* mutants (see Figs 7, S1a). (c) Starch contents of wild-type leaves, *ss4* mutant leaves, and leaves of two lines of *ss4* plants expressing GFP-tagged SS4 at the end of the day (black) and the end of the night (white). Leaf one is the youngest and leaf 16 the oldest leaf. Values are means of measurements on four plants. Error bars are SE.

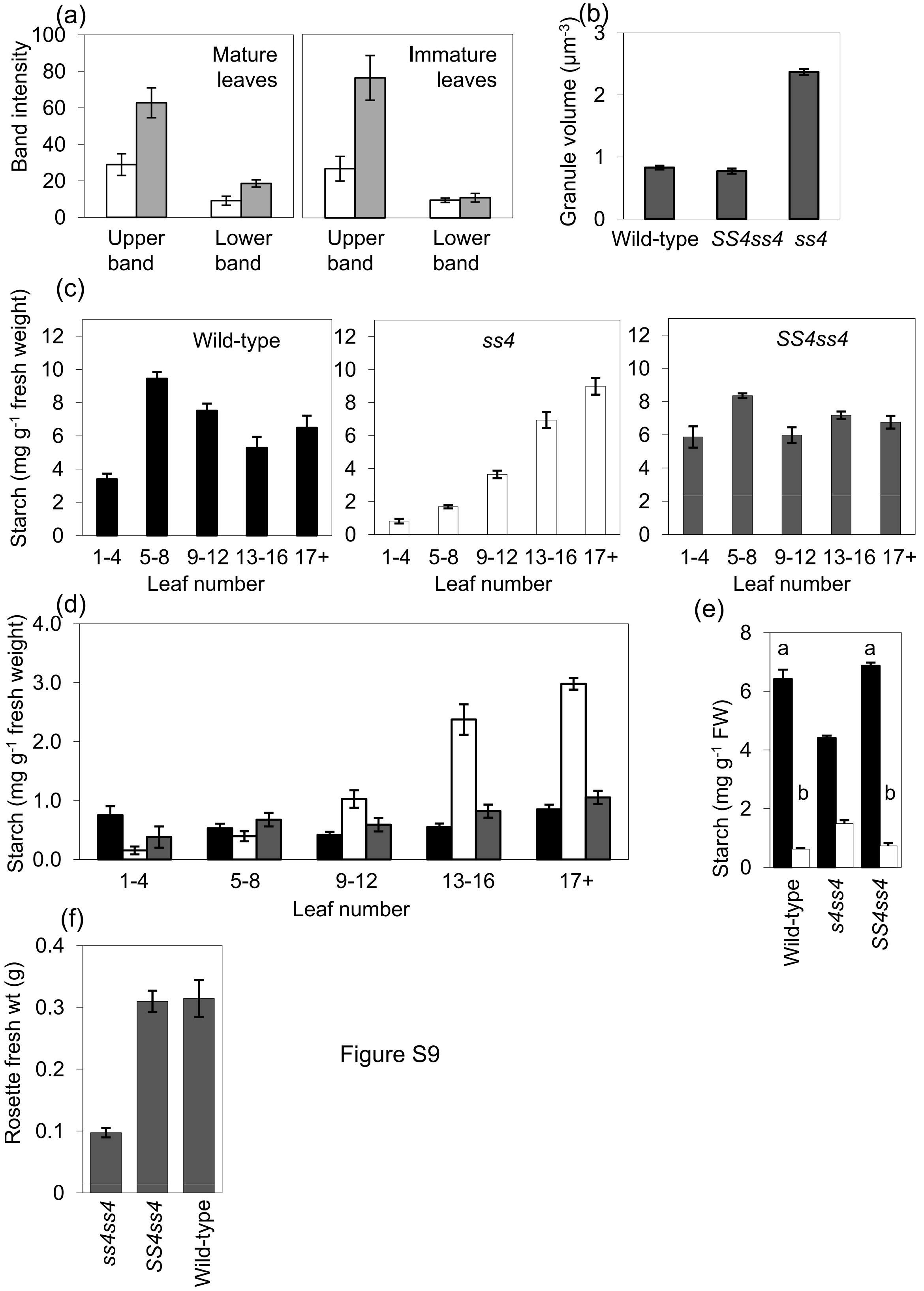
(a)
Band intensity
Mature leaves
Immature leaves
Upper band
Lower band
(b)
Granule volume (µm-3)
Wild-type
SS4ss4
ss4
(c)
Starch (mg g-1 fresh weight)
Wild-type
ss4
SS4ss4
Leaf number
(d)
Starch (mg g-1 fresh weight)
Leaf number
(e)
Starch (mg g-1 FW)
a
b
Wild-type
s4ss4
SS4ss4
(f)
Rosette fresh wt (g)
ss4ss4
SS4ss4
Wild-type

Figure S9

**Fig. S9** Further characterization of heterozygous (*SS4ss4*) plants. (a) Quantification of SS4 upper and lower band intensities from immunoblots as shown in Fig. 7, for extracts of mature leaves and immature leaves. Values (arbitrary units) are means of measurements for three independent heterozygous (white) or wild-type (grey) plants. Error bars are SE. (b) Volumes of granules extracted from wild-type, *SS4ss4* and mutant plants at the end of the day. Radii of between 319 and 2270 granules were measured per genotype, from SEM images, and volumes were calculated using the equations described in Crumpton-Taylor *et al.* (2012). Error bars are SE. (c) Starch contents of leaves of wild-type, *ss4* and *SS4ss4* plants at the end of the day. Leaf one is the youngest and leaf 16 the oldest leaf. Values are means of measurements on at least six rosettes. Error bars are SE. (d) Starch contents of leaves of wild-type (black), *ss4* (white) and *SS4ss4* (grey) plants at the end of the night. Leaf one is the youngest and leaf 16 the oldest leaf. Values are means of measurements on at least six rosettes. Error bars are SE. (e) Starch contents of whole rosettes at the end of the day (black) and the end of the night (white). Values are means of measurements on six rosettes. Error bars are SE. Values with the same letter are not statistically significantly different ($p > 0.05$, Student's t-test). (f) Fresh weights of rosettes 25 d after germination. Values are means of measurements on 15 (*ss4*), 18 (wild-type) or 45 (heterozygote) rosettes. Error bars are SE.
